# Prospection for potential new non-ribosomal peptide gene clusters in *Bacillus* genus isolated from fermented foods and soil through genome mining

**DOI:** 10.3389/fmicb.2025.1515483

**Published:** 2025-06-16

**Authors:** Blaise Waongo, Libère Ndayishimiye, François Tapsoba, Wend-Soo Abel Zongo, Jinquan Li, Aly Savadogo

**Affiliations:** ^1^Laboratory of Applied Biochemistry and Immunology, University Joseph KI-ZERBO, Ouagadougou, Burkina Faso; ^2^National Key Laboratory of Agricultural Microbiology, Key Laboratory of Environment Correlative Dietology, College of Food Science and Technology, College of Biomedicine and Health, Huazhong Agricultural University, Wuhan, China; ^3^College of Food Science and Technology, Zhejiang University of Technology, Hangzhou, China

**Keywords:** *Bacillus*, nonribosomal peptide, lipopeptide, biosynthetic gene clusters, genome mining

## Abstract

Experimental studies, though often very costly, lead to the discovery of known antimicrobial products. Yet, pathogenic microorganisms are proving increasingly resistant to pre-existing antimicrobial molecules, and this is a cause for worldwide concern. Therefore, it is necessary to search for new molecules that could serve as alternatives in the food, medical and agricultural sectors. Thus, 123 complete genomes of *Bacillus* strains isolated from soil and fermented foods were analyzed and annotated using bioinformatics prediction and characterization tools. The view was to discover new gene clusters for the biosynthesis of non-ribosomal peptides (lipopeptides, siderophores, antibiotics). This study revealed that 83% of the genomes analyzed possess biosynthetic gene clusters for the production of siderophore bacillibactin, 61% for surfactins, 37% for fengycins, 23% for iturins, 15% for kurstakins and 3% for bacitracin. Besides, seven new biosynthetic gene clusters coding Non Ribosomal Peptide Synthetases (NRPS) have been identified in *B. velezensis* ATR2, *B. velezensis* DSYZ, *B. velezensis* CGMCC11640, *B. amyloliquefaciens* HM618, *B. amyloliquefaciens* WF02, *B. cereus* CMCC P0011, *B. cereus* CMCC P0021, *B. subtilis* SJ-10 and *B. anthracis* CMF9. The results of this study revealed a significant potential of the genus *Bacillus* to produce new non-ribosomally synthesized peptides. Now, these predicted new antimicrobial molecules can be easily studied experimentally as many new gene clusters have been identified.

## Highlights

- Seven novel biosynthetic gene clusters that encode Non-Ribosomal Peptide Synthetases (NRPS) were discovered.
- A strain of *Bacillus* can potentially co-produce 4 families of lipopeptides.
- *Bacillus* genus has a significant potential to develop new non-ribosomally produced peptides.
- Genome mining strategy makes it possible to discover new metabolites.

## 1 Introduction

*Bacillus* are Gram-positive bacteria belonging to the firmicute genus and *Bacillaceae* family. They are spore-forming, aerobic or facultative aero-anaerobic and get energy by respiration or fermentation (Tamang et al., 2016). Their sporulating ability allows them to resist adverse environmental conditions. They are found in water, soil, dust, plants, food and in animals digestive tract. Some species of the genus *Bacillus* have important roles as antibiotics or antifungals producers (Khurana et al., 2020). For example, locillomycins and surfactin from *B. subtilis* 916 show antibacterial activity against *Xanthomonas oryzae* and *Fusarium oxysporum* (Zhao et al., 2018). Nowadays, 788 species from the genus *Bacillus* are sequenced, leading to various studies and explorations (www.ncbi.nlm.nih.gov/genome/?term=Bacillus, consulted 05/07/2024).

Indeed, many *Bacillus* strains have the ability to produce a wide variety of extracellular enzymes and nonribosomal secondary metabolites such as lipopeptides, the bacillibactin siderophore or antimicrobial compounds such as bacitracin which is a semi-cyclic peptide antibiotic commercialized as a mixture of polypeptides for the treatment of Gram-positive bacterial infections (May et al., 2001; Tapi et al., 2010). Other peptides, such as the lipopeptides have antimicrobial properties and can be used in food or soil biopreservation (Zhao et al., 2018).

*Bacillus* lipopeptides are subdivided into five families (fengycin, iturin, surfactin, kurstakin, and locillomycin) in which various subfamilies and variants can be found (Lam et al., 2021; Waongo et al., 2023). They are produced by modular mega-enzymes or assembly lines referred to as NRPSs (Iqbal et al., 2021). The NRPSs are composed of modules responsible for the incorporation of amino acids in the final nonribosomal peptide (Théatre et al., 2021). The modules contain the following main catalytic domains: the adenylation domain (A), responsible for the amino acid selection and activation, the carrier protein (CP), responsible for tethering the amino acid to the enzyme, and the condensation domain (C), forming a peptide bond between two amino acids attached to two consecutive modules (Leclère et al., 2016). Some NRPSs also include optional domains that can modify the incorporated amino acids during the synthesis, as the Epimerisation domain (E) leading to the D-isomery (Duban et al., 2022). The enzymatic domains work step by step to assemble the monomers into the peptide, so that NRPSs can be compared to assembly lines. A particular trait of NRPSs synthesising Lipopeptides (LP), except those belonging to the iturin and locillomycin families, is the presence of a condensation domain starting the assembly line. The role of this so-called C-starter is to condense the fatty acid into the first amino acid of the peptide chain. The fengycin family includes decapeptides fengycin A, B, C, and plipastatins A, B and S. The iturin family includes bacillomycins (D, DC, F, L and Lc), mycosubtilin, mojavensin A, subtulene A, mixirin and iturins (A, A_L_ and C), all containing 7 amino acids. Heptapeptides are also found in the surfactin family and variant forms of lichenysin, pumilacidin, esperin and kurstakin. The fifth family contains the nonapeptides locillomycins A, B, and C (Luo et al., 2015a; Waongo et al., 2023).

Many drugs remain effective; however, the emergence of resistant organisms has become a major concern, drawing global attention and promoting the “One Health” concept (Aslam et al., 2021). This concept highlights the close links between human, animal, and environmental health. In this context, nonribosomal secondary metabolites produced by the genus *Bacillus* can be a source of interesting active compounds. Indeed, some antimicrobial peptides have been shown to be antibiotics with a broad spectrum against pathogens and lipopeptides and siderophores, which are not antibiotics but represent an alternative for food preservation and plant protection against phytopathogenic fungi (Huan et al., 2020).

An urgent challenge today is to discover new natural drugs to tackle emerging human, animal and plant pathogens. The identification of these biomolecules is usually performed by experimental studies including isolation, characterization, purification and activity tests (Fanaei et al., 2021). With the development of bioinformatics tools, this approach can now be accelerated and complemented by sequencing and subsequent genome or metagenome mining to identify natural product biosynthetic pathways (Blin et al., 2021).

Regarding nonribosomal peptide synthesis, specific tools have been developed to identify biosynthetic gene clusters in the genome sequences, to decipher the organization of NRPS into modules and domains, and to predict the nature of the monomers activated by each Adenylation-domain and their isomery in regard to the presence of epimerization domains (Blin et al., 2023). Notably, bacterial genome mining via *in silico* analysis, using the program antiSMASH, offers an attractive opportunity to discover new secondary metabolites such as NRPs (Rahman et al., 2014; Aleti et al., 2015). AntiSMASH 7 allows better visualization of enzyme assembly chain and good structuring of predicted molecules. Predicted peptides are likely to be characterized by comparison with other peptides available in Norine database (Flissi et al., 2020). The questions addressed here are (i) to review the structural and functional information together with annotation of gene clusters for known lipopeptides (LPs), antibiotics and siderophores produced by the genus *Bacillus* and (ii) to elucidate through genome mining the potential products of yet uncharacterized nonribosomal gene clusters. In addition, a bibliographical search was carried out to better understand the lipopeptides produced by *Bacillus*. The objective of this study is to combine literature exploration and genome analysis to identify and characterize the diversity of nonribosomal peptides as antibiotics, siderophores, and lipopeptides, which are potentially produced by *Bacillus* strains isolated from fermented foods and soil.

## 2 Materials and methods

### 2.1 An overview of known structures of nonribosomal lipopeptides produced by *Bacillus* strains

An overview of known structures of nonribosomal lipopeptides generated by *Bacillus* strains was performed. In brief, Scopus, PubMed, Web of Science, and ResearchGate databases were queried on October 2nd, 2024 using the keywords “lipopeptides AND *Bacillus*, lipopeptides AND structure, surfactin AND *Bacillus*, surfactin AND structure, fengycin AND *Bacillus*, Fengycin AND structure, kurstakin AND *Bacillus*, Kurstakin AND structure, locillomycin AND *Bacillus*, locillomycin AND structure, iturin AND *Bacillus*, iturin AND structure, New lipopeptides AND *Bacillus*, New lipopeptides *Bacillus* AND structure, non-ribosomal peptide AND *Bacillus*.”

### 2.2 Database search for genome sequences

Sequences (including chromosome and plasmid) of *Bacillus* isolated from soil and fermented foods were retrieved from the NCBI nucleotide database.^1^ A total of 123 complete genomes from *Bacillus* strains isolated from soil and fermented food samples, were selected according to the quality (Table 1). Thus, information on assembly, genome size, number of contigs/scaffolds and N50 were examined.

**Table 1:** Genome sequences including chromosomes and plasmids for the different *Bacillus* strains.

Number	Strains	Molecules	Accession number	Isolation source	Country	Molecule size	Contig N50	Completeness (%)	Contamination (%)
1	*B. aerophilus* KJ82	Chromosome	CP091093.1	Soil	China	3,754,440 bp	3.8 Mb	98.92	0.59
2	*B. albus* B-9	Chromosome	NZ_JAAAWQ000000000.1	Soil	China	5,371,297 bp	200.4 kb	99.33	0.1
3	*B. albus* YK87	Chromosome	NZ_CP142670.1	Soil	China	5,347,990 bp	5.3 Mb	98.82	0.10
4	*B. altitudinis* B4133	Chromosome	NZ_JXCN00000000.1	Fermented food	Netherlands	3,718,745 bp	95.4 kb	99.12	0.29
5	*B. altitudinis* GR-8	Chromosome	CP009108.1	Soil	China	3,674,849 bp	3.7 Mb	99.12	0.00
6	*B. altitudinis* GR-8	Plasmid	NZ_CP009109.1	Soil	China	6,935 bp	3.7 Mb	99.12	0.00
7	*B. altitudinis* NJ-V2	Chromosome	NZ_CP012482.1	Soil	China	3,787,818 bp	3.8 Mb	99.12	0.29
8	*B. altitudinis* NJ-M2	Chromosome	NZ_CP012329.1	Soil	China	3,793,419 bp	3.8 Mb	99.12	0.29
9	*B. altitudinis* NJ-V	Chromosome	NZ_CP012330.1	Soil	China	3,792,999 bp	3.8 Mb	99.12	0.29
10	*B. altitudinis* G6S2	Chromosome	NZ_CP126096.1	Soil	Unknown	3,797,173 bp	3.8 Mb	99.41	0.29
11	*B. amyloliquefaciens* S499	Chromosome	CP014700.1	Soil	Congo	3,927,922 bp	3.9 Mb	97.18	0.98
12	*B. amyloliquefaciens* S499	Plasmid	NZ_CP014701.1	Soil	Congo	8,008 bp	3.9 Mb	97.18	0.98
13	*B. amyloliquefaciens* 205	Chromosome	CP054415.1	Soil	China	4,006,790 bp	4 Mb	95.66	1.32
14	*B. amyloliquefaciens* WF02	Chromosome	CP053376.1	Soil	Taiwan	4,026,648 bp	4 Mb	98.24	1.46
15	*B. amyloliquefaciens* bm1	Chromosome	CP088005.1	Soil	China	3,929,792 bp	3.9 Mb	98.03	1.25
16	*B. amyloliquefaciens* LS1-002-014 s	Chromosome	NZ_CP089530.1	Fermented food	China	4,214,996 bp	4.2 Mb	98.26	0.94
17	*B. amyloliquefaciens* HM618	Chromosome	CP029466.1	Soil	China	4,021,851 bp	4 Mb	97.71	0.78
18	*B. amyloliquefaciens* B3	Chromosome	NZ_JAJNPN000000000.1	Fermented food	China	4,001,777 bp	171.8 kb	97.25	2.09
19	*B. anthracis* HYU01	Chromosome	CP008846.1	Soil	South Korea	5,213,498 bp	5.2 Mb	98.09	0.48
20	*B. anthracis* HYU01	Plasmid	CP008847.1	Soil	South Korea	181,894 bp	5.2 Mb	98.09	0.48
21	*B. anthracis* HYU01	Plasmid	CP008848.1	Soil	South Korea	94,732 bp	5.2 Mb	98.09	0.48
22	*B. anthracis* 9080-G	Chromosome	CM002398.1	Soil	Georgia	5,232,192 bp	195.9 kb	98.43	0.71
23	*B. anthracis* 9,080-G	Plasmid	CM002399.1	Soil	Georgia	181,656 bp	195.9 kb	98.43	0.71
24	*B. anthracis* 9,080-G	Plasmid	NZ_CM002400.1	Soil	Georgia	94,815 bp	195.9 kb	98.43	0.71
25	*B. anthracis* CMF9	Chromosome	CP085402.1	Soil	China	5,324,354 bp	5.3 Mb	96.09	3.04
26	*B. arachidis* YX15	Chromosome	CP127376.1	Soil	China	4,913,320 bp	4.9 Mb	98.53	0.00
27	*B. atrophaeus* PENSV20	Chromosome	CP050705.1	Soil	Canada	4,148,820 bp	4.1 Mb	99.12	0.00
28	*B. badius* NBPM-293	Chromosome	CP082363.1	Soil	Unknown	3,868,812 bp	3.9 Mb	98.88	1.16
29	*B. bombysepticus* F12	Chromosome	CP085406.1	Soil	China	5,244,749 bp	5.2 Mb	99.12	0.05
30	*B. bombysepticus* Cuernavaca_S2	Chromosome	CP126590.1	Soil	Mexico	5,288,525 bp	5.3 Mb	98.82	0.00
31	*B. cellulasensis* NJ-V2	Chromosome	CP012482.1	Soil	China	3,787,818 bp	3.8 Mb	99.12	0.29
32	*B. cellulasensis* NJ-M2	Chromosome	CP012329.1	Soil	China	3,793,419 bp	3.8 Mb	99.12	0.29
33	*B. cereus* CMCC P0011	Chromosome	CP011153.1	Soil	China	5,506,876 bp	5.5 Mb	98.97	0.06
34	*B. cereus* CMCC P0011	Plasmid	NZ_CP011154.1	Soil	China	591,112 bp	5.5 Mb	98.97	0.06
35	*B. cereus* M3	Chromosome	CP016316.1	Fermented food	South Korea	5,218,997 bp	5.2 Mb	98.43	0.22
36	*B. cereus* CMCC P0021	Chromosome	CP011151.1	Soil	China	5,521,782 bp	5.5 Mb	99.13	0.06
37	*B. cereus* CMCC P0021	Plasmid	NZ_CP011152.1	Soil	China	591,110 bp	5.5 Mb	99.13	0.06
38	*B. cereus* NJ-W	Chromosome	CP012483.1	Soil	China	5,370,032 bp	5.4 Mb	99.22	0.06
39	*B. cereus* NJ-W	Plasmid	NZ_CP012485.1	Soil	China	11,744 bp	5.4 Mb	99.22	0.06
40	*B. cereus* NJ-W	Plasmid	NZ_CP012484.1	Soil	China	9,032 bp	5.4 Mb	99.22	0.06
41	*B. cereus* NJ-W	Plasmid	NZ_CP012486.1	Soil	China	7,703 bp	5.4 Mb	99.22	0.06
42	*B. cereus* C-1	Chromosome	CP089601.1	Soil	China	5,268,934 bp	5.3 Mb	97.79	0.06
43	*B. gobiensis* FJAT-4402	Chromosome	CP012600.1	Soil	China	4,597,707 bp	2.8 Mb	98.77	1.69
44	*B. haikouensis* MNJ12	Chromosome	CP076017.1	Soil	China	4,454,189 bp	4.5 Mb	99.41	1.78
45	*B. halotolerans* ZB201702	Chromosome	CP029364.1	Soil	China	4,154,245 bp	4.2 Mb	99.41	0.59
46	*B. inaquosorum* DE111	Chromosome	CP013984.1	Soil	USA	4,143,890 bp	4.1 Mb	98.82	0.00
47	B. *inaquosorum* LBA001	Chromosome	NZ_CP127095.1	Soil	Mexico	4,200,707 bp	4.2 Mb	98.82	0.00
48	*B. infantis* 63–11	Chromosome	NZ_JAJBAP020000001.1	Fermented food	Thailand	4,841,671 bp	4.8 Mb	99.41	0.21
49	*B. licheniformis* SCDB 14	Chromosome	CP014842.1	Fermented food	South Korea	4,136,986 bp	4.1 Mb	97.23	1.98
50	*B. licheniformis* P8_B2	Chromosome	CP045814.1	Soil	Denmark	4,343,379 bp	4.3 Mb	97.89	2.24
51	*B. licheniformis* SCK B11	Chromosome	CP014795.1	Soil	South Korea	4,300,706 bp	4.3 Mb	98.33	1.24
52	*B. licheniformis* 14ADL4	Chromosome	CP026673.1	Fermented food	South Korea	4,332,232 bp	4.3 Mb	97.73	3.06
53	*B. methanolicus* MGA3	Chromosome	CP007739.1	Soil	USA	3,337,035 bp	3.3 Mb	97.88	2.66
54	*B. mojavensis* B-41341	Chromosome	NZ_JARLZB000000000.1	Soil	Israel	3,856,892 bp	148 kb	99.41	0.00
55	*B. mojavensis* B-41812	Chromosome	NZ_JARSHG000000000.1	Soil	Argentina	3,924,370 bp	428.5 kb	98.82	0.00
56	*B. mycoides* KBAB4	Chromosome	NC_010184.1	Soil	Unknown	5,262,775 bp	5.3 Mb	98.82	0.32
57	*B. mycoides* KBAB4	Plasmid	NC_010183.1	Soil	Unknown	52,830 bp	5.3 Mb	98.82	0.32
58	*B. mycoides* KBAB4	Plasmid	NC_010182.1	Soil	Unknown	64,977 bp	5.3 Mb	98.82	0.32
59	*B. mycoides* KBAB4	Plasmid	NC_010181.1	Soil	Unknown	75,107 bp	5.3 Mb	98.82	0.32
60	*B. mycoides* BGSC 4BQ1	Chromosome	NZ_NFDI00000000.1	Soil	Spain	5,673,703 bp	639 kb	99.41	0.15
61	*B. mycoides* Gnyt1	Chromosome	CP020743.1	Soil	China	5,597,907 bp	5.6 Mb	99.41	0.00
62	*B. mycoides* ATCC 6462	Chromosome	CP009692.1	Soil	Unknown	5,255,868 bp	5.3 Mb	99.41	0.00
63	*B. pacificus* AT31	Chromosome	NZ_CP142003.1	Soil	China	4,903,194 bp	4.9 Mb	99.41	0.00
64	*B. pacificus* MP6	Chromosome	CP093424.1	Fermented food	China	5,038,982 bp	5 Mb	99.41	0.00
65	*B. pacificus* MP6	Plasmid	CP093425.1	Fermented food	China	291,500 bp	5 Mb	99.41	0.00
66	*B. paralicheniformis* UBBLI-30	Chromosome	NZ_SULF00000000.1	Fermented food	India	4,404,722 bp	332.9 kb	99.41	0.06
67	*B. paralicheniformis* 14DA11	Chromosome	NZ_CP023168.1	Fermented food	South Korea	4,535,069 bp	4.5 Mb	95.35	0.05
68	*B. paralicheniformis* CP47	Chromosome	NZ_CP133705.1	Fermented food	South Korea	4,537,254 bp	4.5 Mb	98.19	0.05
69	*B. paranthracis* SL1	Chromosome	CP093423.1	Fermented food	China	5,243,627 bp	5.2 Mb	99.41	0.29
70	*B. paranthracis* SL1	Plasmid	CP093422.1	Fermented food	China	325,335 bp	5.2 Mb	99.41	0.29
71	*B. paranthracis* Gxun-30	Chromosome	NZ_CP065149.1	Soil	China	5,149,464 bp	5.1 Mb	99.12	0.00
72	*B. paranthracis* KF11	Chromosome	CP085413.1	Soil	China	5,276,246 bp	5.3 Mb	99.41	0.29
73	*B. paranthracis* Bt C4	Chromosome	NZ_CP101135.1	Soil	China	5,245,328 bp	5.2 Mb	98.82	0.29
74	*B. pumilus* NJ-V	Chromosome	CP012330.1	Soil	China	3,792,999 bp	3.8 Mb	99.12	0.29
75	*B. pumilus* DSM 1794	Chromosome	CP187664.1	Soil	Unknown	3,786,030 bp	3.8 Mb	95.31	2.17
76	*B. pumilus* MS32	Chromosome	NZ_CP092829.1	Soil	Germany	3,824,664 bp	3.8 Mb	95.53	2.6
77	*B. pumilus* B4127	Chromosome	NZ_JXCL00000000.1	Fermented food	Netherlands	3,886,280 bp	142 kb	95.37	3.01
78	*B. pumilus* B4127	Plasmid	MH581228.1	Fermented food	Netherlands	6,580 bp	142 kb	95.37	3.01
79	*B. safensis* G6S3	Chromosome	CP128114.1	Soil	Germany	3,670,853 bp	3.7 Mb	99.41	0.29
80	*B. spizizenii* T30	Chromosome	NZ_CP011051.1	Soil	Russia	4,031,727 bp	4 Mb	95.76	0.00
81	*B. spizizenii* AS2	Chromosome	NZ_MUXL00000000.1	Soil	Oman	4,041,230 bp	573.3 kb	98.82	0.29
82	*B. spizizenii* HUK15	Chromosome	NZ_LSMU00000000.1	Soil	France	4,254,363 bp	322.1 kb	98.82	0.29
83	*B. stratosphericus* MRPD-01	Chromosome	PPGB00000000.1	Soil	India	3,687,760 bp	322 kb	99.22	0.29
84	*B. subtilis* SJ-10	Chromosome	NZ_CP025258.1	Fermented food	South Korea	4,041,647 bp	4 Mb	99.36	0.59
85	*B. subtilis* MEC_B298	Chromosome	NZ_CP100436.1	Soil	China	4,030,831 bp	4 Mb	97.41	0.67
86	*B. subtilis* s-16	Chromosome	NZ_CP063150.1	Soil	China	4,209,504 bp	4.2 Mb	97.67	1.35
87	*B. subtilis* BSP1	Chromosome	NZ_CP160396.1	Soil	Lebanon	4,043,759 bp	4 Mb	96.28	1.21
88	*B. subtilis* KH2	Chromosome	CP018184.1	Soil	China	4,138,265 bp	4.1 Mb	96.73	1.28
89	*B. subtilis* FUA2231	Chromosome	NZ_CP154918.1	Fermented food	Zimbabwe	4,475,230 bp	4.5 Mb	97.31	1.62
90	*B. subtilis* UD1022	Chromosome	CP011534.1	Soil	USA	4,025,326 bp	4 Mb	97.53	0.91
91	*B. subtilis* SFA-H43	Chromosome	KZ836066.1	Soil	China	4,018,162 bp	2.1 Mb	96.73	1.48
92	*B. subtilis* CGMCC 2108	Chromosome	CP014471.1	Soil	China	4,122,154 bp	4.1 Mb	96.73	1.08
93	*B. subtilis* CGMCC 2108	Plasmid	NZ_CP014473.1	Soil	China	65,774 bp	4.1 Mb	96.73	1.08
94	*B. subtilis* CGMCC 2108	Plasmid	NZ_CP014472.1	Soil	China	5,820 bp	4.1 Mb	96.73	1.08
95	*B. subtilis* T30	Chromosome	CP011051.1	Soil	Russia	4,031,727 bp	4 Mb	95.76	0.00
96	*B. subtilis* NCIB 3610	Chromosome	NZ_CP094361.1	Soil	China	4,210,909 bp	4.2 Mb	98.51	1.33
97	*B. subtilis* ZD01	Chromosome	NZ_CP046448.1	Soil	China	4,015,360 bp	4 Mb	97.5	0.67
98	*B. subtilis* 73	Chromosome	CP045826.1	Soil	Germany	4,166,516 bp	4.2 Mb	97.33	1.97
99	*B. subtilis* G8	Chromosome	NZ_AP025224.1	Fermented food	Indonesia	4,017,503 bp	4 Mb	96.73	1.08
100	*B. subtilis* FUA2232	Chromosome	NZ_CP154920.1	Fermented food	Zimbabwe	4,475,228 bp	4.5 Mb	97.31	1.61
101	*B. subtilis* SRCM103517	Chromosome	NZ_CP035226.1	Fermented food	South Korea	4,192,706 bp	4.2 Mb	96.85	1.87
102	*B. subtilis* S1	Chromosome	NZ_JAGFPW000000000.1	Fermented food	India	4,488,261 bp	153.3 kb	95.77	1.85
103	*B. thuringiensis* HD12	Chromosome	CP014847.1	Soil	China	5,776,895 bp	5.8 Mb	98.39	0.96
104	*B. thuringiensis* HD12	Plasmid	NZ_CP014853.1	Soil	China	345,196 bp	5.8 Mb	98.39	0.96
105	*B. thuringiensis* HD12	Plasmid	NZ_CP014852.1	Soil	China	161,353 bp	5.8 Mb	98.39	0.96
106	*B. thuringiensis* HD12	Plasmid	NZ_CP014851.1	Soil	China	112,429 bp	5.8 Mb	98.39	0.96
107	*B. thuringiensis* HD12	Plasmid	NZ_CP014850.1	Soil	China	39,023 bp	5.8 Mb	98.39	0.96
108	*B. thuringiensis* HD12	Plasmid	NZ_CP014848.1	Soil	China	17,228 bp	5.8 Mb	98.39	0.96
109	*B. thuringiensis* Bt185	Chromosome	CP014282.1	Soil	China	5,243,635 bp	5.2 Mb	98.26	1.05
110	*B. thuringiensis* Bt185	Plasmid	NZ_CP014284.1	Soil	China	293,705 bp	5.2 Mb	98.26	1.05
111	*B. thuringiensis* Bt185	Plasmid	NZ_CP014285.1	Soil	China	55,372 bp	5.2 Mb	98.26	1.05
112	*B. thuringiensis* Bt185	Plasmid	NZ_CP014286.1	Soil	China	54,205 bp	5.2 Mb	98.26	1.05
113	*B. thuringiensis* Bt185	Plasmid	NZ_CP014287.1	Soil	China	41,937 bp	5.2 Mb	98.26	1.05
114	*B. thuringiensis* Bt185	Plasmid	NZ_CP014288.1	Soil	China	12,487 bp	5.2 Mb	98.26	1.05
115	*B. thuringiensis* Bt185	Plasmid	NZ_CP014290.1	Soil	China	7,486 bp	5.2 Mb	98.26	1.05
116	*B. thuringiensis* c25	Chromosome	NZ_CP022345.1	Soil	South Korea	5,334,660 bp	5.3 Mb	98.91	1.07
117	*B. thuringiensis* 97–27	Chromosome	NC_005957.1	Soil	Unknown	5,237,682 bp	5.2 Mb	99.44	0.32
118	*B. thuringiensis* YWC2-8	Chromosome	CP013055.1	Soil	China	5,674,369 bp	5.7 Mb	98.38	1.01
119	*B. thuringiensis* YWC2-8	Plasmid	NZ_CP013056.1	Soil	China	250,706 bp	5.7 Mb	98.38	1.01
120	*B. thuringiensis* YWC2-8	Plasmid	NZ_CP013057.1	Soil	China	84,491 bp	5.7 Mb	98.38	1.01
121	*B. thuringiensis* YWC2-8	Plasmid	NZ_CP013058.1	Soil	China	82,531 bp	5.7 Mb	98.38	1.01
122	*B. thuringiensis* YWC2-8	Plasmid	NZ_CP013059.1	Soil	China	80,699 bp	5.7 Mb	98.38	1.01
123	*B. thuringiensis* YWC2-8	Plasmid	NZ_CP013060.1	Soil	China	46,634 bp	5.7 Mb	98.38	1.01
124	*B. thuringiensis* YWC2-8	Plasmid	NZ_CP013061.1	Soil	China	8,512 bp	5.7 Mb	98.38	1.01
125	*B. thuringiensis* BGSC 4AA1	Chromosome	CP010577.1	Soil	China	5,652,292 bp	5.7 Mb	97.24	3.40
126	*B. thuringiensis* BGSC 4AA1	Plasmid	NZ_CP010578.1	Soil	China	232,994 bp	5.7 Mb	97.24	3.40
127	*B. thuringiensis* BGSC 4AA1	Plasmid	NZ_CP010579.1	Soil	China	92,619 bp	5.7 Mb	97.24	3.40
128	*B. thuringiensis* BGSC 4AA1	Plasmid	NZ_CP010580.1	Soil	China	76,979 bp	5.7 Mb	97.24	3.40
129	*B. thuringiensis* BGSC 4AA1	Plasmid	NZ_CP010581.1	Soil	China	68,444 bp	5.7 Mb	97.24	3.40
130	*B. thuringiensis* BGSC 4AA1	Plasmid	NZ_CP010582.1	Soil	China	51,723 bp	5.7 Mb	97.24	3.40
131	*B. thuringiensis* BGSC 4AA1	Plasmid	NZ_CP010583.1	Soil	China	4,845 bp	5.7 Mb	97.24	3.40
132	*B. thuringiensis* CTC	Chromosome	CP013274.1	Soil	China	5,327,397 bp	5.3 Mb	97.63	1.10
133	*B. thuringiensis* CTC	Plasmid	NZ_CP013273.1	Soil	China	25,529 bp	5.3 Mb	97.63	1.10
134	*B. thuringiensis* HS18-1	Chromosome	CP012099.1	Soil	China	5,292,526 bp	5.3 Mb	98.68	0.55
135	*B. thuringiensis* HS18-1	Plasmid	NZ_CP012101.1	Soil	China	337,579 bp	5.3 Mb	98.68	0.55
136	*B. thuringiensis* HS18-1	Plasmid	NZ_CP012102.1	Soil	China	92,085 bp	5.3 Mb	98.68	0.55
137	*B. thuringiensis* HS18-1	Plasmid	NZ_CP012103.1	Soil	China	94,695 bp	5.3 Mb	98.68	0.55
138	*B. thuringiensis* HS18-1	Plasmid	NZ_CP012104.1	Soil	China	42,726 bp	5.3 Mb	98.68	0.55
139	*B. thuringiensis* HS18-1	Plasmid	NZ_CP012105.1	Soil	China	14,336 bp	5.3 Mb	98.68	0.55
140	*B. thuringiensis* HS18-1	Plasmid	NZ_CP012106.1	Soil	China	4,669 bp	5.3 Mb	98.68	0.55
141	*B. thuringiensis* HS18-1	Plasmid	NZ_CP012107.1	Soil	China	8,287 bp	5.3 Mb	98.68	0.55
142	*B. thuringiensis* HS18-1	Plasmid	NZ_CP012108.1	Soil	China	7,386 bp	5.3 Mb	98.68	0.55
143	*B. thuringiensis* JW-1	Chromosome	NZ_CP045030.1	Soil	China	5,500,376 bp	5.5 Mb	99.30	0.74
144	*B. thuringiensis* HD-1	Chromosome	CP004870.1	Soil	USA	5,631,672 bp	5.6 Mb	97.67	1.75
145	*B. thuringiensis* YBT-1518	Chromosome	CP005935.1	Soil	China	6,002,284 bp	6 Mb	98.60	3.18
146	*B. toyonensis* UTDF19-29B	Chromosome	CP081872.1	Soil	USA	5,240,743 bp	5.2 Mb	99.41	0.10
147	*B. toyonensis* Monterrey_S3	Chromosome	CP126524.1	Soil	Mexico	5,309,620 bp	5.3 Mb	99.41	0.10
148	*B. toyonensis* Cuernavaca_S4	Chromosome	CP126520.1	Soil	Mexico	5,270,039 bp	5.3 Mb	98.82	0.10
149	*B. tropicus* CK18	Chromosome	CP085399.1	Soil	China	5,237,233 bp	5.2 Mb	99.41	0.00
150	*B. tropicus* T36S-23	Chromosome	NZ_CP119875.1	Soil	South Korea	5,262,398 bp	5.3 Mb	98.82	0.62
151	*B. vallismortis* NBIF-001	Chromosome	CP020893.1	Soil	China	3,929,787 bp	3.9 Mb	98.82	0.00
152	*B. vallismortis* DSM 11031	Chromosome	CP026362.1	Soil	USA	4,286,362 bp	4.3 Mb	99.22	0.00
153	*B. velezensis* ATR2	Chromosome	CP018133.1	Soil	China	4,006,746 bp	4 Mb	99.41	0.00
154	*B. velezensis* SYP-B637	Chromosome	CP043546.1	Soil	China	3,915,550 bp	3.9 Mb	99.41	0.00
155	*B. velezensis* CGMCC 11640	Chromosome	CP026610.1	Soil	China	4,322,979 bp	4.3 Mb	99.41	0.00
156	*B. velezensis* Lzh-a42	Chromosome	CP025308.1	Soil	China	4,246,605 bp	4.2 Mb	99.41	0.00
157	*B. velezensis* YJ0-1	Chromosome	NZ_CP128184.1	Soil	China	4,043,341 bp	4 Mb	99.41	1.24
158	*B. velezensis* L-S60	Chromosome	CP011278.1	Soil	China	3,903,017 bp	3.9 Mb	99.41	0.00
159	*B. velezensis* CBMB205	Chromosome	CP014838.1	Soil	South Korea	3,929,745 bp	3.9 Mb	99.41	0.00
160	*B. velezensis* SB1216	Chromosome	CP015417.1	Soil	USA	3,814,720 bp	3.8 Mb	97.56	0.00
161	*B. velezensis* DSYZ	Chromosome	CP030150.1	Soil	China	4,258,978 bp	4.3 Mb	99.41	0.00
162	*B. velezensis* DR-08	Chromosome	CP028437.1	Soil	South Korea	3,929,794 bp	3.9 Mb	99.36	0.00
163	*B. velezensis* CMF18	Chromosome	CP085388.1	Soil	China	3,963,155 bp	4 Mb	99.41	0.00
164	*B. velezensis* CK17	Chromosome	CP085706.1	Soil	China	3,921,806 bp	3.9 Mb	99.34	0.00
165	*B. velezensis* DMB06	Chromosome	NZ_CP083763.1	Fermented food	South Korea	4,157,945 bp	4.2 Mb	99.4	0.00
166	*B. velezensis* UD6-2	Chromosome	NZ_JAGFMB000000000.1	Fermented food	Thailand	3,951,373 bp	1 Mb	99.41	0.00
167	*B. velezensis* DMB05	Chromosome	NZ_CP083715.1	Fermented food	South Korea	3,262,563 bp	3.3 Mb	99.12	0.00
168	*B. velezensis* DMB05	Plasmid	NZ_CP083716.1	Fermented food	South Korea	806,695 bp	3.3 Mb	99.12	0.00
169	*B. velezensis* DMB05	Plasmid	NZ_CP083717.1	Fermented food	South Korea	72,020 bp	3.3 Mb	99.12	0.00
170	*B. wiedmannii* LN15	Chromosome	CP126099.1	Soil	China	5,391,143 bp	5.4 Mb	98.82	0.10
171	*B. wiedmannii* JAS08/1	Chromosome	NZ_CP036073.1	Soil	Poland	5,513,922 bp	5.5 Mb	99.41	0.10
172	*B. wiedmannii* JAS07/5	Chromosome	CP036070.1	Soil	Poland	5,239,489 bp	5.2 Mb	99.41	1.76
173	*B. wiedmannii* JAS07/5	Plasmid	NZ_CP036072.1	Soil	Poland	536,465 bp	5.2 Mb	99.41	1.76
174	*B. wiedmannii* JAS07/5	Plasmid	NZ_CP036071.1	Soil	Poland	79,335 bp	5.2 Mb	99.41	1.76
175	*B. wiedmannii* PL1	Chromosome	AP022643.1	Soil	Japan	5,309,441 bp	5.3 Mb	97.22	0.00
176	*B. safensis* ZK-1	Chromosome	NZ_CP095759.1	Soil	China	3,735,476 bp	3.7 Mb	99.06	0.29
177	*B. safensis* SRCM125915	Chromosome	NZ_CP116774.1	Soil	South Korea	3,769,976 bp	3.8 Mb	98.92	0.59
178	*B. safensis* PLA 1006	Chromosome	NZ_CP114177.1	Soil	China	3,891,304 bp	3.9 Mb	98.92	0.59

### 2.3 Detection of nonribosomal peptide biosynthetic gene clusters (BGCs)

The search for nonribosomal peptide sequences from the genome sequences of 123 *Bacillus* strains was performed following a workflow previously described (Leclère et al., 2016). First, the prediction of biosynthetic gene clusters (BGCs) was performed using antiSMASH version 7 (Blin et al., 2023). The regions encoding nonribosomal synthetases were further analyzed. The monomer composition of the predicted peptide was compared to all known NRPs annotated in the Norine database^2^ (Flissi et al., 2020) in order to identify them or predict them as a new peptide or new variant. When it was incomplete with truncated or fragmented clusters, we further investigated antiSMASH results to reconstruct partial or complete BGCs by assembling cluster fragments scattered in the shotgun genome. The number of genes, the order of modules, and the domains in the NRPSs and the predictions of the A-domain specificity helped identify clusters of known NRP families, even when they were fragmented.

Since poor sequence assembly can affect the quality of predicted metabolites, only high-quality complete genomes were considered in this study.

## 3 Results

Out of 123 *Bacillus* chromosomes selected, 115 were found to carry BGC NRPSs. Among these NRPSs, those responsible for the synthesis of a siderophore (102), antibiotics (4) or lipopeptides (93) were found.

### 3.1 Prediction of NRP siderophore

The screened sequences harbor genetic potential to produce the siderophore bacillibactin. The gene cluster is composed of three modules with A-domain specificity for 2,3-dihydroxybenzoate (Dhb or diOH-Bz in Norine), glycine and threonine. The corresponding NRPS is known to follow an iterative mode of biosynthesis, which could lead to the production of active iron-chelating compounds named bacillibactins. This small cluster was predicted in 83% of the studied genomes.

### 3.2 Overview of lipopeptide BGCs produced by *Bacillus*

The literature search revealed diverse non-ribosomal lipopeptides synthesized by the genus *Bacillus*. The structures of the different molecules are shown in Table 2.

**Table 2:** Structure of different lipopeptides classified by families.

Name of lipopeptides	Monomer composition of the peptide moiety	Main fatty acid chains	References
Fengycin family	Decapeptide with a lactone ring between carboxy-terminal group	β-OH fatty acids	
Fengycin A1	L-Glu,D-Orn,D-Tyr,D-aThr,L-Glu,D-Ala,L-Pro,L-Gln,L-Tyr,L-Ile	C_14_-C_19_	Ait Kaki et al. (2020)
Fengycin A2	L-Glu,D-Orn,D-Tyr,D-aThr,L-Glu,D-Ala,L-Pro,L-Gln,L-Tyr,L-Val	C_14_, C_15_, C_17_	Ongena and Jacques (2007)
Fengycin B1	L-Glu-D-Orn-D-Tyr-D-aThr,L-Glu,D-Val,L-Pro,L-Gln,L-Tyr,L-Ile	C_14_-C_17_	Fanaei et al. (2021)
Fengycin B2	L-Glu,D-Orn,D-Tyr,D-aThr,L-Glu,D-Val,L-Pro,L-Gln,L-Tyr,L-Val	C_14_-C_21_	Ongena and Jacques (2007)
Fengycin C1	L-Glu,D-Orn,D-Tyr,D-aThr,L-Glu,D-Leu/D-Ile,L-Pro,L-Gln,L-Tyr,L-Val	C_15_-C_19_	Vater et al. (2002)
Fengycin C2	L-Glu,D-Orn,D-Tyr,D-aThr,L-Glu,D-Val,L-Pro,L-Gln,L-aThr,L-Ile	C_14_-C_18_	Ongena and Jacques (2007)
Fengycin S	L-Glu,D-Orn,D-Tyr,D-Ser,L-Glu,D-Val,L-Pro,L-Gln,L-Tyr,L-Ile	C_17_	Sang-cheol et al. (2010)
Plipastatin A1, A2	L-Glu,D-Orn,D-Tyr,D-a-Thr,L-Glu,D-Ala,L-Pro,L-Gln,D-Tyr,L-Ile	nC_16_, aiC_17_	Nishikiori et al. (1986)
Plipastatin B1, B2	L-Glu,D-Orn,D-Tyr,D-a-Thr,L-Glu,D-Val,L-Pro,L-Gln,L-Tyr,L-Ile	nC_16_, aiC_17_	Nishikiori et al. (1986)
Iturin family	Heptapeptide cyclised by a lactam ring with the β-NH2 group of the acid chain	β-NH_2_ fatty acids	
Iturin A, A_L_	L-Asn,D-Tyr,D-Asn,L-Gln,L-Pro,D-Asn,L-Ser	nC_14_, iC_15_, aiC_15_, nC_16_, iC_16_	Pathak and Keharia (2014)
Iturin C	L-Asp,D-Tyr,D-Asn,L-Gln,L-Pro,D-Asn,L-Ser	nC_14_, iC_15_, aiC_15_	Pathak and Keharia (2014)
Bacillomycin D	L-Asn,D-Tyr,D-Asn,L-Pro,L-Gln,D-Ser,L-Thr	nC_14_, iC_15_, aiC_15_	Jin et al. (2017)
Bacillomycin DC	L-Asn,D-Tyr,D-Asn,L-Pro,L-Glu,D-Ser,L-Thr	C_13_	Jin et al. (2017)
Bacillomycin F	L-Asn,D-Tyr,D-Asn,L-Gln,L-Pro,D-Asn,L-Thr	C_16_, iC_17_, aiC_17_	Peypoux et al. (1985)
Bacillomycin L	L-Asp,D-Tyr,D-Asn,L-Ser,L-Glu,D-Ser,L-Thr	nC_14_, iC_15_, aiC_15_	Volpon et al. (2007)
Bacillomycin Lc^a^	L-Asn,D-Tyr,D-Asn,L-Ser,L-Glu,D-Ser,L-Thr	nC_14_, iC_15_, aiC_15_, iC_16_	Volpon et al. (2007)
Mycosubtilin	L-Asn,D-Tyr,D-Asn,L-Gln,L-Pro,D-Asn/D-Ser,L-Ser/L-Asn	nC_16_, iC_16_, aiC_17_	Peypoux et al. (1986)
Mojavensin A	L-Asp,D-Tyr,D-Asn,L-Gln,L-Pro,D-Asn,L-Asn	aiC_12_	Ma et al. (2012)
Subtulene A	L-Asn,D-Tyr,D-Asn,L-Gln,L-Pro,D-Asn,L-Ser	(CH2)4HC = CH(CH2)3CH(CH3)2	Thasana et al. (2010)
Mixirin	L-Asn,D-Tyr,D-Asn,L-Gln,L-Ser,D-Asn,L-Pro	(A) C_11_, (B) C_8_, (C) *ai*C_10_	Zhang et al. (2004)
Kurstakin family	Heptapeptide with a lactone ring between carboxy-terminal group of Gln7 and OH group of Ser4		
Kurstakin^b^	D-Thr,L-Gly,D-Ala,L-Ser,L-His,D-Gln,L-Gln	iC_11_, nC_12_, iC_12_, iC_13_	Hathout et al. (2000)
Surfactin family	Heptapeptide closed by a lactone ring with the b-OH group of the fatty acid chain	β-OH fatty acids	
Bamylocin A^c^	Glu,Leu,Met,Leu,Pro,Leu,Leu,Leu	C_13_	Lee et al. (2007)
Surfactin	L-Glu,L-Leu,D-Leu,L-Val,L-Asp,D-Leu,L-Leu	iC_14_, nC_14_, iC_15_, aC_15_	Bonmatin et al. (2003)
[Orn2] surfactin	L-Glu,L-Orn,D-Leu,L-Val,L-Asp,D-Leu,L-Ile	C_13_-C_15_	Bonmatin et al. (2003)
[Orn7] surfactin	L-Glu,L-Leu,D-Leu,L-Val,L-Asp,D-Leu,L-Orn	–	Bonmatin et al. (2003)
[Phe7] surfactin	L-Glu,L-Leu,D-Leu,L-Val,L-Asp,D-Leu,L-Phe	–	Bonmatin et al. (2003)
[Cys7] surfactin	L-Glu,L-Leu,D-Leu,L-Val,L-Asp,D-Leu,L-Cys	–	Bonmatin et al. (2003)
[Ala4] surfactin	L-Glu,L-Leu,D-Leu,L-Ala,L-Asp,D-Leu,L-Leu	iC_14_, nC_14_, iC_15_, aiC_15_	Bonmatin et al. (2003)
[Ile4] surfactin	L-Glu,L-Leu,D-Leu,L-Ile,L-Asp,D-Leu,L-Leu	aiC_15_	Bonmatin et al. (2003)
[Ile2, 7] surfactin	L-Glu,L-Ile,D-Leu,L-Val,L-Asp,D-Leu,L-Ile	C_13_-C_15_	Bonmatin et al. (2003)
[Ile4, 7] surfactin	L-Glu,L-Leu,D-Leu,L-Ile,L-Asp,D-Leu,L-Ile	C_13_-C_15_	Bonmatin et al. (2003)
[Leu4] surfactin	L-Glu,L-Leu,D-Leu,L-Leu,L-Asp,D-Leu,L-Leu	iC_15_	Bonmatin et al. (2003)
[Val7] surfactin	L-Glu,L-Leu,D-Leu,L-Val,L-Asp,D-Leu,L-Val	iC_14_, nC_14_, iC_15_, aiC_15_	Bonmatin et al. (2003)
[Ile7] surfactin	L-Glu,L-Leu,D-Leu,L-Val,L-Asp,D-Leu,L-Ile	iC_14_, nC_14_, iC_15_, aiC_15_	Bonmatin et al. (2003)
[Ile2, 4] surfactin	L-Glu,L-Ile,D-Leu,L-Ile,L-Asp,D-Leu,L-Leu	aiC_15_	Bonmatin et al. (2003)
[Val2, 7] surfactin	L-Glu,L-Val,D-Leu,L-Val,L-Asp,D-Leu,L-Val	C_13_-C_15_	Bonmatin et al. (2003)
[Val2, Ile7] surfactin	L-Glu,L-Val,D-Leu,L-Val,L-Asp,D-Leu,L-Ile	C_13_-C_15_	Bonmatin et al. (2003)
[Ile2, Val7] surfactin	L-Glu,L-Ile,D-Leu,L-Val,L-Asp,D-Leu,L-Val	C_13_-C_15_	Bonmatin et al. (2003)
[Ile2, 4, 7] surfactin	L-Glu,L-Ile,D-Leu,L-Ile,L-Asp,D-Leu,L-Ile	aiC_15_	Bonmatin et al. (2003)
Lichenysin^e^	L-Gln,L-Leu,D-Leu,L-Val,L-Asp,D-Leu,L-Ile	iC_13_, aC_13_, nC_14_ iC_15_, aC_15_	Bonmatin et al. (2003)
[Ile4] lichenysin	L-Gln,L-Leu,D-Leu,L-Ile,L-Asp,D-Leu,L-Ile	aiC_15_	Bonmatin et al. (2003)
[Val7] lichenysin	L-Gln,L-Leu,D-Leu,L-Val,L-Asp,D-Leu,L-Val	iC_13_, aiC_13_, nC_14_, iC_15_, aiC_15_	Bonmatin et al. (2003)
[Ile2, 4] lichenysin	L-Gln,L-Ile,D-Leu,L-Ile,L-Asp,D-Leu,L-Ile	aiC_15_	Bonmatin et al. (2003)
Esperin^d^	L-Glu,L-Leu,D-Leu,L-Val,L-Asp,D-Leu,L-XE7-COOH	C_13_, C_14_, C_15_	Thomas and Ito (1969)
Pumilacidin	L-Glu,L-Leu,D-Leu,L-Leu,L-Asp,D-Leu,L-XP7	aC_15_, iC_15_, nC_16_, iC_16_, aC_17_, iC_17_	Naruse et al. (1990)
Locillomycin family	Partially cyclized nonapeptides		
Locillomycin A, B, C	L-Thr,D-Gln,L-Asp,L-Gly,L-Asn,L-Asp,L-Gly,L-Tyr,L-Val	C_13_, C_14_, C_15_	Luo et al. (2015a, 2015b)
Cerexin family	Linear decapeptides		
Cerexin A	D-Asp,D-Val,D-val,L-Asn,D-Asn,L-_ϒ_Hyl,D-Thr,L-Ser,D-Trp,D-Ile	iC_11_h^3^	Shoji et al. (1976b)
Cerexin B	D-Asn,D-val,D-Phe,L-Asn,D-Asn,L-_ϒ_Hyl,D-Thr,L-Gly,D-Trp,D-Ile	iC_10_h^3^, nC_10_h^3^, iC_11_h^3^, aiC_11_h^3^	Shoji and Kato (1976)
Cerexin C	D-Asp,D-Val,D-val,L-Asn,D-Asn,L-Lys,D-Thr,L-Ser,D-Trp,D-Ile	iC_11_h^3^	Shoji et al. (1976a)
Cerexin D	D-Asp,D-Val,D-Phe,L-Asn,D-Asn,L-Lys,D-Thr,L-Gly,D-Trp,D-Ile	iC_10_h^3^, nC_10_h^3^, iC_11_h^3^, aiC_11_h^3^	Shoji et al. (1976a)
Marihysin A family	Cyclic heptapeptide		
Marihysin A	Pro,Gln,Asn,Ser,Asn,Tyr,Asn	C_14_	Liu et al. (2010)
Octapeptin family			
Octapeptin A1, A2, A3, A4	D-Dab,L-Dab,L-Dab,L-Dab,L-Dab,D-Leu,L-Leu,L-Leu	aiC_11_h^3^, iC_10_h^3^, nC_10_h^3^, iC_11_h^3^	Meyers et al. (1976)
Octapeptin B1, B2, B3, B4	D-Dab,L-Dab,L-Dab,L-Dab,L-Dab,D-Leu,L-Leu,L-Phe	aiC_11_h^3^, iC_10_h^3^, nC_10_h^3^, iC_9_h^3^	Meyers et al. (1976)
Octapeptin C1, C2, C3, C4	D-Dab,L-Dab,L-Dab,L-Dab,L-Dab,L-Leu,L-Leu,D-Phe	aiC_9_h^3^, aiC_9_h^3^, iC_10_h^3^, nC_10_h^3^	Meyers et al. (1976)
Octapeptin D1, D2, D3, D4	D-Ser-L-Dab-L-Dab-D-Leu-L-Leu-L-Dab-L-Dab-L-Leu	aiC_11_h^3^, iC_10_h^3^, nC_10_h^3^, iC_11_h^3^	Kato and Shoji (1980)

^a^Bacillomycin Lca; or bacillopeptin.

^b^D forms of amino acid residue are deduced from NRPS modular structure.

^c^L and D forms are not specified.

^d^The b-carboxyl of Asp5 is engaged in the lactone.

^e^Or halobacillin With XE7 = Leu or Val; XP7 = Val or Ile.

ai, anteiso; i, iso; n, normal; h^3^, 3-hydroxy; L-Hyl, L-threo—hydroxylysine; Dab, 2,4-Diaminobutyric acid.

### 3.3 Prediction of the lipopeptide structures

**Figure 1:**
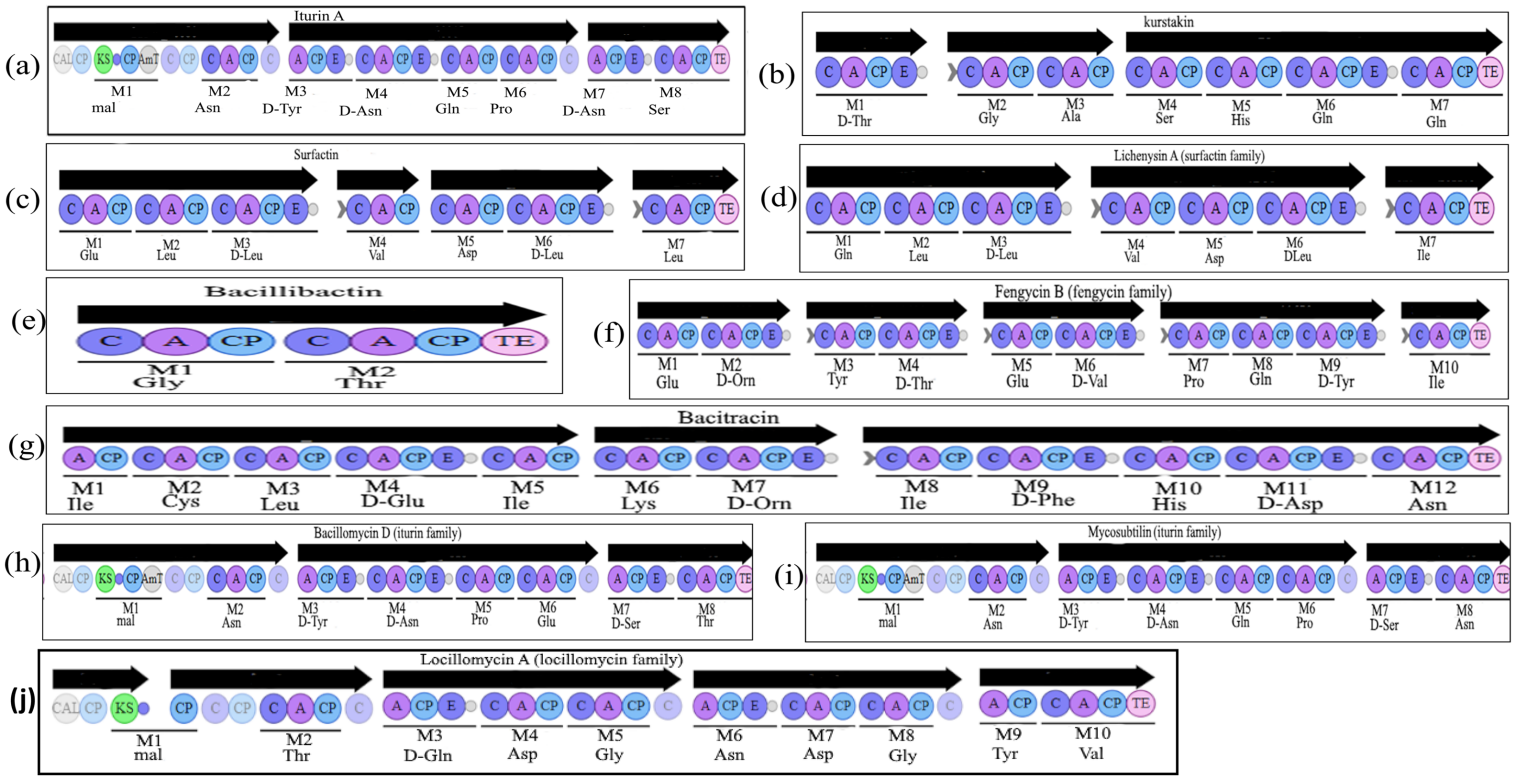
Organization of the known non-ribosomal peptide synthetases (NRPS) encoding lipopeptides, siderophore bacillibactin and antibiotic bacitracin in *Bacillus*. Iterative domains: A, adenylation domain; C, condensation domain; CP, Carrier Protein; E, epimerization domain; TE, thioesterase domain; mal, malonyl-CoA; CAL, Co-enzyme A ligase; KS, ketosynthetase domain; AmT, aminotransferase. Organization of the known NRPSs: **(a)** iturin A in *B. amyloliquefaciens* S499, **(b)** known kurstakin, **(c)** surfactin in *B. velezensis* UD6-2, **(d)** lichenysin A in *B. paralicheniformis* CP47, **(e)** bacillibactin in *B. aerophilus* KJ82, **(f)** fengycin B in *B. paralicheniformis* CP47, **(g)** bacitracin in *B. paralicheniformis* CP47, **(h)** bacillomycin D in *B. velezensis* DMB06, **(i)** mycosubtilin in *B. subtilis* T30, **(j)** locillomycin in *B. subtilis* 916.

Within BGCs detected by antiSMASH, special attention was paid to traits specific to LP synthesis. Thus, a condensation domain allowing the incorporation of fatty acid (FA) (so-called C-starter) was sought. C-starter is usually present in LP NRPS except for LP belonging to iturin and locillomycin families (Figure 1). As up to now, no bioinformatics tool enables the prediction of fatty acid (FA) structure, the work was focused on peptide moieties (Table 3).

**Table 3:** Structures of predicted and identified lipopeptides among different genomic sequences of *Bacillus* strains.

Predicted LP	Structure analysis	Comments	Strains
[Ile7] surfactin	(Glu,Leu,D-Leu)(Val,Asp,D-Leu)(Ile)	Complete	*B. aerophilus* KJ82
[Ile7] surfactin	(Glu,Leu,D-Leu)(Val,Asp,Leu)(Ile)	Complete	*B. altitudinis* B4133
[Ile7] surfactin	(Glu,Leu,D-Leu)(Val,Asp,D-Leu)(Ile)	Complete	*B. altitudinis* GR-8
[Ile7] surfactin	(Glu,Leu,D-Leu)(Val,Asp,D-Leu)(Ile)	Complete	*B. altitudinis* NJ-V2
[Ile7] surfactin	(Glu,Leu,D-Leu)(Val,Asp,D-Leu)(Ile)	Complete	*B. altitudinis* NJ-M2
[Ile7] surfactin	(Glu,Leu,D-Leu)(Val,Asp,D-Leu)(Ile)	Complete	*B. altitudinis* NJ-V
[Ile7] surfactin	(Glu,Leu,D-Leu)(Val,Asp,D-Leu)(Ile)	Complete	*B. altitudinis* G6S2
Iturin A	(Asn)(D-Tyr,D-Asn,Gln,Pro)(D-Asn,Ser)	Complete	*B. amyloliquefaciens* S499
Fengycin A/B	(Glu,D-Orn)(Tyr,**D-Thr**)(Glu,D-X)(Pro,Gln,D-Tyr)(Ile)	Incomplete (X could be Val or Ala)	*B. amyloliquefaciens* S499
Surfactin	(Glu,Leu,D-Leu)(…)(Leu)	Partial and fragmented	*B. amyloliquefaciens* S499
[Ile7] surfactin	(Glu,Leu,D-Leu)(Val,Asp,D-Leu)(Ile)	Complete	*B. amyloliquefaciens* 205
Fengycin	(……)(…)(…)(…Glu,D-Tyr)(Ile)	Partial and fragmented (Glu instead of Gln)	*B. amyloliquefaciens* 205
Iturin A	(Asn)(D-Tyr,D-Asn,Gln,Pro)(D-Asn,Ser)	Complete	*B. amyloliquefaciens* 205
Surfactin	(Glu,Leu,D-Leu)(Val,Asp,D-Leu)(Leu)	Complete	*B. amyloliquefaciens* WF02
Fengycin A/B	(Glu,D-Orn)(Tyr,**D-Thr**)(Glu,D-X)(Pro,Gln,D-Tyr)(Ile)	Incomplete (X could be Val or Ala)	*B. amyloliquefaciens* WF02
Iturin A	(Asn)(D-Tyr,D-Asn,Gln,Pro)(D-Asn,Ser)	Complete	*B. amyloliquefaciens* WF02
Surfactin	(Glu,Leu,D-Leu)(Val,Asp,Leu)(Leu)	Complete	*B. amyloliquefaciens* bm1
Fengycin A/B	(Glu,D-Orn,Tyr,**D-Thr**)(Glu,D-X)(Pro,Gln,D-Tyr)(Ile)	Incomplete (X could be Val or Ala)	*B. amyloliquefaciens* bm1
Iturin A	(Asn)(D-Tyr,D-Asn,Gln,Pro)(D-Asn,Ser)	Complete	*B. amyloliquefaciens* bm1
Surfactin	(Glu,Leu,D-Leu)(Val,Asp,D-Leu)(Leu)	Complete	*B. amyloliquefaciens* LS1-002-014 s
Fengycin A/B	(Glu,D-Orn)(Tyr,**D-Thr**)(Glu,D-X)(Pro,Gln,D-Tyr)(Ile)	Incomplete (X could be Val or Ala)	*B. amyloliquefaciens* LS1-002-014 s
Iturin A	(Asn)(D-Tyr,D-Asn,Gln,Pro)(D-Asn,Ser)	Complete	*B. amyloliquefaciens* LS1-002-014 s
Surfactin	(Glu,Leu,D-Leu)(Val,Asp,D-Leu)(Leu)	Complete	*B. amyloliquefaciens* HM618
Fengycin	(……)(Tyr,**D-Thr**)(Glu,D-X)(Pro,Gln,D-Tyr)(Ile)	Partial (X could be Val or Ala)	*B. amyloliquefaciens* HM618
Iturin A	(Asn)(D-Tyr,D-Asn,Gln,Pro)(D-Asn,Ser)	Complete	*B. amyloliquefaciens* HM618
Surfactin	(Glu,Leu,…)(Val,Asp,…)(Leu)	Partial and fragmented	*B. amyloliquefaciens* B3
Fengycin	(……)(…)(…,D-X)(Pro,Gln,D-Tyr)(Ile)	Partial	*B. amyloliquefaciens* B3
[Ile7] surfactin	(Glu,Leu,D-Leu)(Val,Asp,D-Leu)(Ile)	Complete	*B. atrophaeus* PENSV20
Fengycin A/B	(Glu,D-Orn)(Tyr,**D-Thr**)(Glu,D-X)(Pro,Gln,D-Tyr)(Ile)	Incomplete (X could be Val or Ala)	*B. atrophaeus* PENSV20
Iturin A	(Asn)(D-Tyr,D-Asn,Gln,Pro)(…,Ser)	Partial	*B. atrophaeus* PENSV20
Kurstakin	(D-Thr)(X,Ala)(Ser,Leu,D-X,Glu)	Incomplete (Leu instead of His; Glu instead of Gln; X, X could be Gly and Gln respectively)	*B. bombysepticus* F12
Kurstakin	(D-Thr)(X,Ala)(Ser,Leu,D-X,Glu)	Incomplete (Leu instead of His; Glu instead of Gln; X, X could be Gly and Gln respectively)	*B. bombysepticus* Cuernavaca_S2
[Ile7] surfactin	(Glu,Leu,D-Leu)(Val,Asp,D-Leu)(Ile)	Complete	*B. cellulasensis* NJ-V2
[Ile7] surfactin	(Glu,Leu,D-Leu)(Val,Asp,D-Leu)(Ile)	complete	*B. cellulasensis* NJ-M2
Kurstakin	(D-Thr)(X,Ala)(Ser,Leu,D-X,Glu)	Incomplete (Leu instead of His; Glu instead of Gln; X, X could be Gly and Gln respectively)	*B. cereus* CMCC P0011
Kurstakin	(D-Thr)(X,Ala)(Ser,Leu,D-X,Glu)	Incomplete (Leu instead of His; Glu instead of Gln; X, X could be Gly and Gln respectively)	*B. cereus* CMCC P0021
Kurstakin	(D-Thr)(X,Ala)(Ser,Leu,D-X,Glu)	Incomplete (Leu instead of His; Glu instead of Gln; X, X could be Gly and Gln respectively)	*B. cereus* NJ-W
Kurstakin	(D-Thr)(X,Ala)(Ser,Leu,D-X,Glu)	Incomplete (Leu instead of His; Glu instead of Gln; X, X could be Gly and Gln respectively)	*B. cereus* C-1
Surfactin	(Glu,Leu,D-Leu)(Val,Asp,D-Leu)(Leu)	Complete	*B. halotolerans* ZB201702
Fengycin	(Glu,D-Orn)(Tyr,**D-Thr**)(Glu,D-X)(Pro,Glu,…)(…)	Partial (Glu instead of Gln and X could be Val or Ala)	*B. halotolerans* ZB201702
Fengycin	(…,D-Orn)(Tyr,**D-Thr**)(…,…)(Pro,…,…)(Ile)	Partial and fragmented	*B. inaquosorum* DE111
Surfactin	(Glu,Leu,D-Leu)(Val,Asp,…)(Leu)	Partial and fragmented	*B. inaquosorum* DE111
Surfactin	(Glu,Leu,D-Leu)(Val,Asp,D-Leu)(Leu)	Complete	*B. inaquosorum* LBA001
Fengycin	(…,D-Orn)(Tyr,**D-Thr**)(Glu,…)(Pro,Glu,…)(Ile)	Partial and fragmented (Glu instead of Gln)	*B. inaquosorum* LBA001
Bacillomycin F	(Asn)(D-Tyr,D-Asn,Pro,Gln)(D-Asn,Thr)	Complete	*B. inaquosorum* LBA001
Lichenysin A	(Gln,Leu,D-Leu)(Val,Asp,D-Leu)(Ile)	Complete	*B. licheniformis* SCDB 14
Lichenysin A	(Gln,Leu,D-Leu)(Val,Asp,D-Leu)(Ile)	Complete	*B. licheniformis* P8_B2
Lichenysin A	(Gln,Leu,D-Leu)(Val,Asp,D-Leu)(Ile)	Complete	*B. licheniformis* SCK B11
Surfactin	(Glu,Leu,D-Leu)(Val,Asp,D-Leu)(Leu)	Complete	*B. licheniformis* 14ADL4
Fengycin	(…)(…)(…,Glu,D-Tyr)(Ile)	Partial and fragmented	*B. licheniformis* 14ADL4
Fengycin	(…,D-Orn)(Tyr,**D-Thr**)(…,D-X)(Pro,Glu,D-Tyr)(Ile)	Partial and fragmented (Glu instead of Gln and X could be Val)	*B. mojavensis* B-41341
Surfactin	(Glu,Leu,…)(Val,Asp,…)(Leu)	Partial and fragmented	*B. mojavensis* B-41341
Surfactin	(Glu,Leu,D-Leu)(Val,Asp,D-Leu)(Leu)	Complete	*B. mojavensis* B-41812
Fengycin B variant	(Glu,D-Orn)(Tyr,**D-Thr**)(Glu,D-X)(Pro,Glu,D-Tyr)(Ile)	Incomplete (Glu instead of Gln and X could be Val)	*B. mojavensis* B-41812
Kurstakin variant	(D-Thr)(X,Ala)(Ser,Leu,D-Thr,Glu)	Incomplete (Leu instead of His; Thr instead of Gln; Glu instead of Gln; X could be Gly)	*B. mycoides* BGSC 4BQ1
Kurstakin	(D-Thr)(X,Ala)(Ser,Leu,D-X,Glu)	Incomplete (Leu instead of His; Glu instead of Gln; X, X could be Gly and Gln respectively)	*B. mycoides* Gnyt1
Lichenysin A	(Gln,Leu,D-Leu)(Val,Asp,…)(Ile)	Partial and fragmented	*B. paralicheniformis* UBBLI-30
Fengycin B	(…,D-Orn)(Tyr,**D-Thr**)(…,D-Val)(Pro,Gln,D-Tyr)(Ile)	Partial and fragmented	*B. paralicheniformis* UBBLI-30
Lichenysin A	(Gln,Leu,D-Leu)(Val,Asp,D-Leu)(Ile)	Complete	*B. paralicheniformis* 14DA11
Fengycin B	(Glu,…)(Tyr,**D-Thr**)(Glu,Val)(Pro,Gln,D-Tyr)(Ile)	Partial and fragmented	*B. paralicheniformis* 14DA11
Lichenysin A	(Gln,Leu,D-Leu)(Val,Asp,D-Leu)(Ile)	Complete	*B. paralicheniformis* CP47
Fengycin B	(Glu,D-Orn)(Tyr,**D-Thr**)(Glu,D-Val)(Pro,Gln,D-Tyr)(Ile)	Complete	*B. paralicheniformis* CP47
[Ile7] surfactin	(Glu,Leu,D-Leu)(Val,Asp,D-Leu)(Ile)	Complete	*B. pumilus* NJ-V
[Ile7] surfactin	(Glu,Leu,D-Leu)(Val,Asp,D-Leu)(Ile)	Complete	*B. pumilus* DSM 1794
[Ile7] surfactin	(Glu,Leu,D-Leu)(Val,Asp,D-Leu)(Ile)	Complete	*B. pumilus* MS32
[Ile7] surfactin	(Glu,Leu,D-Leu)(Val,Asp,…)(Ile)	Partial	*B. pumilus* B4127
[Ile4,7] surfactin	(Glu,Leu,D-Leu)(Ile,Asp,D-Leu)(Ile)	Complete	*B. safensis* G6S3
Surfactin	(Glu,Leu,D-Leu)(Val,Asp,Leu)(Leu)	Complete	*B. spizizenii* T30
Mycosubtilin	(Asn)(D-Tyr,D-Asn,Gln,Pro)(D-Ser,Asn)	Complete	*B. spizizenii* T30
Mycosubtilin	(Asn)(D-Tyr,D-Asn,Gln,Pro)(D-Ser,Asn)	Complete	*B. spizizenii* AS2
Surfactin	(Glu,Leu,…)(Val,Asp,…)(Leu)	Partial and fragmented	*B. spizizenii* AS2
Mycosubtilin	(Asn)(D-Tyr,D-Asn,Gln,Pro)(D-Ser,Asn)	Complete	*B. spizizenii* HUK15
Surfactin	(Glu,Leu,…)(Val,Asp,…)(Leu)	Partial and fragmented	*B. spizizenii* HUK15
Surfactin	(Glu,Leu,D-Leu)(Val,Asp,D-Leu)(Leu)	Complete	*B. subtilis* SJ-10
Fengycin B	(Glu,D-Orn)(Tyr,**D-Thr**)(Glu,D-Val)(Pro,Glu,D-Tyr)(Ile)	Complete	*B. subtilis* SJ-10
Bacillomycin D	(Asn)(D-Tyr,D-Asn,Pro,Glu)(D-Ser,Thr)	Complete	*B. subtilis* SJ-10
Surfactin	(Glu,Leu,D-Leu)(Val,Asp,D-Leu)(Leu)	Complete	*B. subtilis* MEC_B298
Fengycin B variant	(…,…)(Tyr,**D-Thr**)(Glu,D-Val)(Pro,Glu,D-Tyr)(Ile)	Partial (Glu instead of Gln)	*B. subtilis* MEC_B298
Surfactin	(Glu,Leu,D-Leu)(Val,Asp,D-Leu)(Leu)	Complete	*B. subtilis* s-16
Fengycin B variant	(Glu,…)(Tyr**,D-Thr**)(Glu,D-Val)(Pro,Glu,D-Tyr)(Ile)	Partial and fragmented (Glu instead of Gln)	*B. subtilis* s-16
Fengycin B variant	(Glu,…)(Tyr,**D-Thr**)(Glu,D-Val)(Pro,Glu,D-Tyr)(Ile)	Partial and fragmented (Glu instead of Gln)	*B. subtilis* BSP1
Surfactin	(Glu,Leu,D-Leu)(Val,Asp,D-Leu)(Leu)	Complete	*B. subtilis* BSP1
Surfactin	(Glu,Leu,D-Leu)(Val,Asp,D-Leu)(Leu)	Complete	*B. subtilis* KH2
Surfactin	(Glu,Leu,D-Leu)(Val,Asp,D-Leu)(Leu)	Complete	*B. subtilis* FUA2231
Fengycin B variant	(Glu,D-Orn,Tyr,**D-Thr**)(Glu,D-Val)(Pro,Glu,D-Tyr)(Ile)	Complete (Glu instead of Gln)	*B. subtilis* FUA2231
Surfactin	(Glu,Leu,D-Leu)(Val,Asp,D-Leu)(Leu)	Complete	*B. subtilis* UD1022
Fengycin B variant	(Glu,D-Orn)(Tyr,**D-Thr**)(Glu,D-Val)(Pro,Glu,D-Tyr)(Ile)	Complete (Glu instead of Gln)	*B. subtilis* UD1022
Surfactin	(Glu,Leu,D-Leu)(Val,Asp,D-Leu)(Leu)	Complete	*B. subtilis* SFA-H43
Surfactin	(Glu,Leu,D-Leu)(Val,Asp,D-Leu)(Leu)	Complete	*B. subtilis* CGMCC 2108
Mycosubtilin	(Asn)(D-Tyr,D-Asn,Gln,Pro)(D-Ser,Asn)	Complete	*B. subtilis* T30
Surfactin	(Glu,Leu,D-Leu)(Val,Asp,D-Leu)(Leu)	Complete	*B. subtilis* T30
Surfactin	(Glu,Leu,D-Leu)(Val,Asp,D-Leu)(Leu)	Complete	*B. subtilis* NCIB 3610
Fengycin	(Glu,D-Orn)(Tyr,**D-Thr**)(…,…)(Pro,Glu,D-Tyr)(Ile)	Partial and fragmented (Glu instead of Gln)	*B. subtilis* NCIB 3610
Fengycin B variant	(Glu,D-Orn)(Tyr,**D-Thr**)(Glu,D-Val)(Pro,Glu,D-Tyr)(Ile)	Complete (Glu instead of Gln)	*B. subtilis* ZD01
Surfactin	(Glu,Leu,D-Leu)(Val,Asp,D-Leu)(Leu)	Complete	*B. subtilis* ZD01
Surfactin	(Glu,Leu,D-Leu)(Val,Asp,D-Leu)(Leu)	Complete	*B. subtilis* 73
Fengycin	(…)(Glu,D-Tyr)(Ile)	Partial and fragmented	*B. subtilis* 73
[Ile7] surfactin	(Glu,Leu,…)(Val,Asp,…)(Ile)	Partial and fragmented	*B. subtilis* G8
Fengycin	(…)(…)(…,Glu,D-Tyr)(…)	Partial and fragmented	*B. subtilis* G8
Surfactin	(Glu,Leu,D-Leu)(Val,Asp,D-Leu)(Leu)	Complete	*B. subtilis* FUA2232
Fengycin B variant	(Glu,D-Orn,Tyr,**D-Thr**)(Glu,D-Val)(Pro,Glu,D-Tyr)(Ile)	Complete (Glu instead of Gln)	*B. subtilis* FUA2232
Surfactin	(Glu,Leu, D-Leu)(Val,Asp,D-Leu)(Leu)	Complete	*B. subtilis* SRCM103517
Fengycin B variant	(Glu,D-Orn,Tyr,**D-Thr**)(Glu,D-Val)(Pro,Glu,D-Tyr)(Ile)	Complete (Glu instead of Gln)	*B. subtilis* SRCM103517
Surfactin	(Glu,Leu,…)(Val,Asp,…)(Leu)	Partial and fragmented	*B. subtilis* S1
Kurstakin	(D-Thr)(X,Ala)(Ser,Leu,D-X,Glu)	Incomplete (Leu instead of His; Glu instead of Gln; X, X could be Gly and Gln respectively)	*B. thuringiensis* HD12
Kurstakin	(D-Thr)(X,Ala)(Ser,Leu,D-X,Glu)	Incomplete (Leu instead of His; Glu instead of Gln; X, X could be Gly and Gln respectively)	*B. thuringiensis* Bt185
Kurstakin	(D-Thr)(X,Ala)(Ser,Leu,D-X,Glu)	Incomplete (Leu instead of His; Glu instead of Gln; X, X could be Gly and Gln respectively)	*B. thuringiensis* YWC2-8
Kurstakin	(D-Thr)(X,Ala)(Ser,Leu,D-X,Glu)	Incomplete (Leu instead of His; Glu instead of Gln; X, X could be Gly and Gln respectively)	*B. thuringiensis* BGSC 4AA1
Kurstakin	(D-Thr)(X,Ala)(Ser,Leu,D-X,Glu)	Incomplete (Leu instead of His; Glu instead of Gln; X, X could be Gly and Gln respectively)	*B. thuringiensis* HS18-1
Kurstakin	(D-Thr)(X-Ala)(Ser,Leu,D-X,Glu)	Incomplete (Leu instead of His; Glu instead of Gln; X, X could be Gly and Gln respectively)	*B. thuringiensis* c25
Kurstakin	(D-Thr)(X-Ala)(Ser,Leu,D-X,Glu)	Incomplete (Leu instead of His; Glu instead of Gln; X, X could be Gly and Gln respectively)	*B. thuringiensis* JW-1
Kurstakin	(D-Thr)(X,Ala)(Ser,Leu,D-X,Glu)	Incomplete (Leu instead of His; Glu instead of Gln; X, X could be Gly and Gln respectively)	*B. thuringiensis* HD-1
Kurstakin	(D-Thr)(X,Ala)(Ser,Leu,D-X,Glu)	Incomplete (Leu instead of His; Glu instead of Gln; X, X could be Gly and Gln respectively)	*B. thuringiensis* YBT-1518
Surfactin	(Glu,Leu,D-Leu)(Val,Asp,D-Leu)(Leu)	Complete	*B. vallismortis* NBIF-001
Fengycin A/B	(Glu,D-Orn)(Tyr,**D-Thr**)(Glu,D-X)(Pro,Gln,D-Tyr)(Ile)	Incomplete (X Could be Val or Ala)	*B. vallismortis* NBIF-001
Iturin A	(Asn)(D-Tyr,D-Asn,Gln,Pro)(D-Asn,Ser)	Complete	*B. vallismortis* NBIF-001
Fengycin B variant	(Glu,D-Orn)(Tyr,**D-Thr**)(Glu,D-Val)(Pro,Glu,D-Tyr)(Ile)	Complete (Glu instead of Gln)	*B. vallismortis* DSM 11031
Surfactin	(Glu,Leu,D-Leu)(Val,Asp,D-Leu)(Leu)	Complete	*B. vallismortis* DSM 11031
Surfactin	(Glu,Leu,D-Leu)(Val,Asp,D-Leu)(Leu)	Complete	*B. velezensis* ATR2
Fengycin	(Glu,D-Orn)(Tyr,**D-Thr**)(……)(Pro,Gln…)(Ile)	Partial and fragmented	*B. velezensis* ATR2
Bacillomycin D	(Asn)(D-Tyr,D-Asn,Pro,Glu)(D-Ser,Thr)	Complete	*B. velezensis* ATR2
Surfactin	(Glu,Leu,D-Leu)(Val,Asp,D-Leu)(Leu)	Complete	*B. velezensis* SYP-B637
Fengycin A/B	(Glu,D-Orn)(Tyr,**D-Thr**)(Glu,D-X)(Pro,Gln,D-Tyr)(Ile)	Incomplete (X could be Val or Ala)	*B. velezensis* SYP-B637
Bacillomycin D	(Asn)(D-Tyr,D-Asn,Pro,Glu)(D-Ser,Thr)	Complete	*B. velezensis* SYP-B637
Surfactin	(Glu,Leu,D-Leu)(Val,Asp,D-Leu)(Leu)	Complete	*B. velezensis* CGMCC 11640
Fengycin A/B	(Glu,D-Orn)(Tyr,**D-Thr**)(Glu,D-X)(Pro,Gln,D-Tyr)(Ile)	Incomplete (X could be Val or Ala)	*B. velezensis* CGMCC 11640
Iturin A	(Asn)(D-Tyr,D-Asn,Gln,Pro)(D-Asn,Ser)	Complete	*B. velezensis* CGMCC 11640
Surfactin	(Glu,Leu,D-Leu)(Val,Asp,…)(Leu)	Partial and fragmented	*B. velezensis* Lzh-a42
Fengycin A/B	(Glu,D-Orn)(Tyr,**D-Thr**)(Glu,D-X)(Pro,Gln,D-Tyr)(Ile)	Incomplete (X could be Val or Ala)	*B. velezensis* Lzh-a42
Bacillomycin D	(Asn)(D-Tyr,D-Asn,Pro,Glu)(D-Ser,Thr)	Complete	*B. velezensis* Lzh-a42
Fengycin A/B	(Glu,D-Orn)(Tyr,**D-Thr**)(Glu,D-X)(Pro,Gln,D-Tyr)(Ile)	Incomplete (X could be Val or Ala)	*B. velezensis* YJ0-1
Bacillomycin D	(Asn)(D-Tyr,D-Asn,Pro,Glu)(D-Ser,Thr)	Complete	*B. velezensis* YJ0-1
Surfactin	(Glu,Leu,D-Leu)(Val)(Asp,D-Leu)(Leu)	Complete	*B. velezensis* YJ0-1
Surfactin	(Glu,Leu,D-Leu)(Val,Asp,D-Leu)(Leu)	Complete	*B. velezensis* L-S60
Fengycin	(Glu,D-Orn)(Tyr,**D-Thr**)(……)(Pro,Gln,D-Tyr)(Ile)	Partial and fragmented	*B. velezensis* L-S60
Iturin A	(Asn)(D-Tyr,D-Asn,Gln,Pro)(D-Asn,Ser)	Complete	*B. velezensis* L-S60
Surfactin	(Glu,Leu,D-Leu)(Val,Asp,D-Leu)(Leu)	Complete	*B. velezensis* CBMB205
Fengycin A/B	(Glu,D-Orn)(Tyr,**D-Thr**)(Glu,D-X)(Pro,Gln,D-Tyr)(Ile)	Incomplete (X could be Val or Ala)	*B. velezensis* CBMB205
Iturin A	(Asn)(D-Tyr,D-Asn,Gln,Pro)(D-Asn,Ser)	Complete	*B. velezensis* CBMB205
Surfactin	(Glu,Leu,D-Leu)(Val,Asp,D-Leu)(Leu)	Complete	*B. velezensis* SB1216
Bacillomycin L	(Asn)(D-Tyr,D-Asn,Ser,Glu)(D-Ser,Thr)	Complete	*B. velezensis* SB1216
Fengycin	(………)(Tyr,**D-Thr**)(……)(Pro,……)(Ile)	Partial and fragmented	*B. velezensis* SB1216
Surfactin	(Glu,Leu,…)(Val,Asp,D-Leu)(Leu)	Partial and fragmented	*B. velezensis* DSYZ
Fengycin A/B	(Glu,D-Orn)(Tyr,**D-Thr**)(Glu,D-X)(Pro,Gln,D-Tyr)(Ile)	Incomplete (X could be Val or Ala)	*B. velezensis* DSYZ
Iturin A	(Asn)(D-Tyr,D-Asn,Gln,Pro)(D-Asn,Ser)	Complete	*B. velezensis* DSYZ
Surfactin	(Glu,Leu,D-Leu)(Val,Asp,D-Leu)(Leu)	Complete	*B. velezensis* DR-08
Fengycin A/B	(Glu,D-Orn)(Tyr,**D-Thr**)(Glu,D-X)(Pro,Gln,D-Tyr)(Ile)	Incomplete (X could be Val or Ala)	*B. velezensis* DR-08
Iturin A	(Asn)(D-Tyr,D-Asn,Gln,Pro)(D-Asn,Ser)	Complete	*B. velezensis* DR-08
Surfactin	(Glu,Leu,D-Leu)(Val,Asp,D-Leu)(Leu)	Complete	*B. velezensis* CMF18
Fengycin A/B	(Glu,D-Orn)(Tyr,**D-Thr**)(Glu,D-X)(Pro,Gln,D-Tyr)(Ile)	Incomplete (X could be Val or Ala)	*B. velezensis* CMF18
Iturin A	(Asn)(D-Tyr,D-Asn,Gln,Pro)(D-Asn,Ser)	Complete	*B. velezensis* CMF18
Surfactin	(Glu,Leu,D-Leu)(Val,Asp,D-Leu)(Leu)	Complete	*B. velezensis* CK17
Fengycin A/B	(Glu,D-Orn)(Tyr,**D-Thr**)(Glu,D-X)(Pro,Gln,D-Tyr)(Ile)	Incomplete (X could be Val or Ala)	*B. velezensis* CK17
Iturin A	(Asn)(D-Tyr,D-Asn,Gln,Pro)(D-Asn,Ser)	Complete	*B. velezensis* CK17
Surfactin	(Glu,Leu,D-Leu)(Val,Asp,D-Leu)(Leu)	Complete	*B. velezensis* DMB06
Bacillomycin D	(Asn)(D-Tyr,D-Asn,Pro,Glu)(D-Ser,Thr)	Complete	*B. velezensis* DMB06
Fengycin	(………)(…)(…,D-X)(Pro,…)(Ile)	Partial and fragmented	*B. velezensis* DMB06
Surfactin	(Glu,Leu,D-Leu)(Val,Asp,D-Leu)(Leu)	Complete	*B. velezensis* UD6-2
Fengycin	(……)(…)(…,D-X)(Pro…)(Ile)	Partial and fragmented	*B. velezensis* UD6-2
Bacillomycin D	(Asn)(D-Tyr,D-Asn,Pro,Glu)(D-Ser,Thr)	Complete	*B. velezensis* UD6-2
Surfactin	(Glu,Leu,D-Leu)(Val,Asp,D-Leu)(Leu)	Complete	*B. velezensis* DMB05
Fengycin	(……)(……)(…X)(Pro,…)(Ile)	Partial and fragmented	*B. velezensis* DMB05
Bacillomycin D	(Asn)(D-Tyr,D-Asn,Pro,Glu)(D-Ser,Thr)	Complete	*B. velezensis* DMB05
Kurstakin	(D-Thr)(X,Ala)(Ser,Leu,D-X,Glu)	Incomplete (Leu instead of His; Glu instead of Gln; X, X could be Gly and Gln respectively)	*B. wiedmannii* JAS07/5
Kurstakin	(D-Thr)(X,Ala)(Ser,Leu,D-X,Glu)	Incomplete (Leu instead of His; Glu instead of Gln; X, X could be Gly and Gln respectively)	*B. wiedmannii* PL1
[Ile4,7] surfactin	(Glu,Leu,D-Leu)(Ile,Asp,D-Leu)(Ile)	Complete	*B. safensis* ZK-1
[Ile4,7] surfactin	(Glu,Leu,D-Leu)(Ile,Asp,D-Leu)(Ile)	Complete	*B. safensis* SRCM125915
[Ile4,7] surfactin	(Glu,Leu,D-Leu)(Ile,Asp,D-Leu)(Ile)	Complete	*B. safensis* PLA 1006

The monomers in brackets are those synthesized by a single gene. Monomers in bold differ from the known variants described in the same position. When the gene cluster was incomplete because scattered on several contigs or located on a too small contig, it was specify in the comment column and symbolized the truncated genes by “…”.

**Figure 2:**
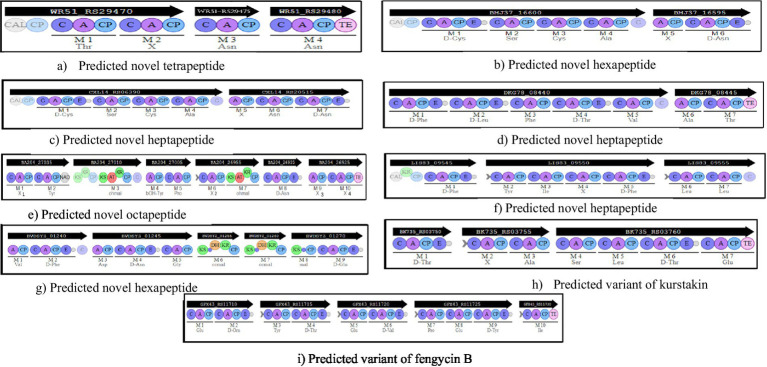
Organization of the predicted novel NRPSs and NRPS/PKS. Iterative domains: A, adenylation domain; C, condensation domain; CP, Carrier Protein; E, epimerization domain; TE, thioesterase domain; AT, acetyltransferase domain; KS, ketosynthetase domain; KR, ketoreductase domain; CAL, Coenzyme A ligase; C surrounded in light blue, condensation domain allowing condensation between a PKS monomer and a NRPS monomer. Predicted amino acid specificity is shown under each A domain. **(a)** a tetrapeptide in *B. cereus* CMCCP0011 (plasmid) and *B. cereus* CMCCP021 (plasmid); **(b)** a hexapeptide in *B. velezensis* ATR2 (chromosome); **(c)** a heptapeptide in *B. velezensis* DSYZ (chromosome), *B. subtilis* SJ-10 (chromosome) and *B. velezensis* CGMCC 11640 (chromosome); **(d)** a heptapeptide in *B. amyloliquefaciens* HM618 (chromosome); **(e)** a octapeptide in *B. cereus* CMCC P0011 (plasmid) and *B. cereus* CMCC P0021 (plasmid); **(f)** a heptapeptide in *B. anthracis* CMF9 (chromosome); **(g)** a hexapeptide in *B. amyloliquefaciens* WF02 (chromosome), *B. velezensis* CGMCC 11640 (chromosome) and *B. velezensis* DSYZ (chromosome); **(h)** predicted variant of kurstakin in *B. mycoide* BGSC 4BQ1, the difference with the other kurstakin predicted concerning the composition amino acids is located at the level of monomer 6; **(i)** predicted variant of fengycin B in *B. vallismortis* DSM 11031, *B. subtilis* UD1022, *B. subtilis* FUA2231, *B. subtilis* FUA2232, *B. subtilis* SRCM103517 and *B. subtilis* ZD01 genomes. Modular architecture is similar to the known fengycin B, the difference with fengycin B regarding the composition in amino acids is located at the level monomer 8.

**Figure 3:**
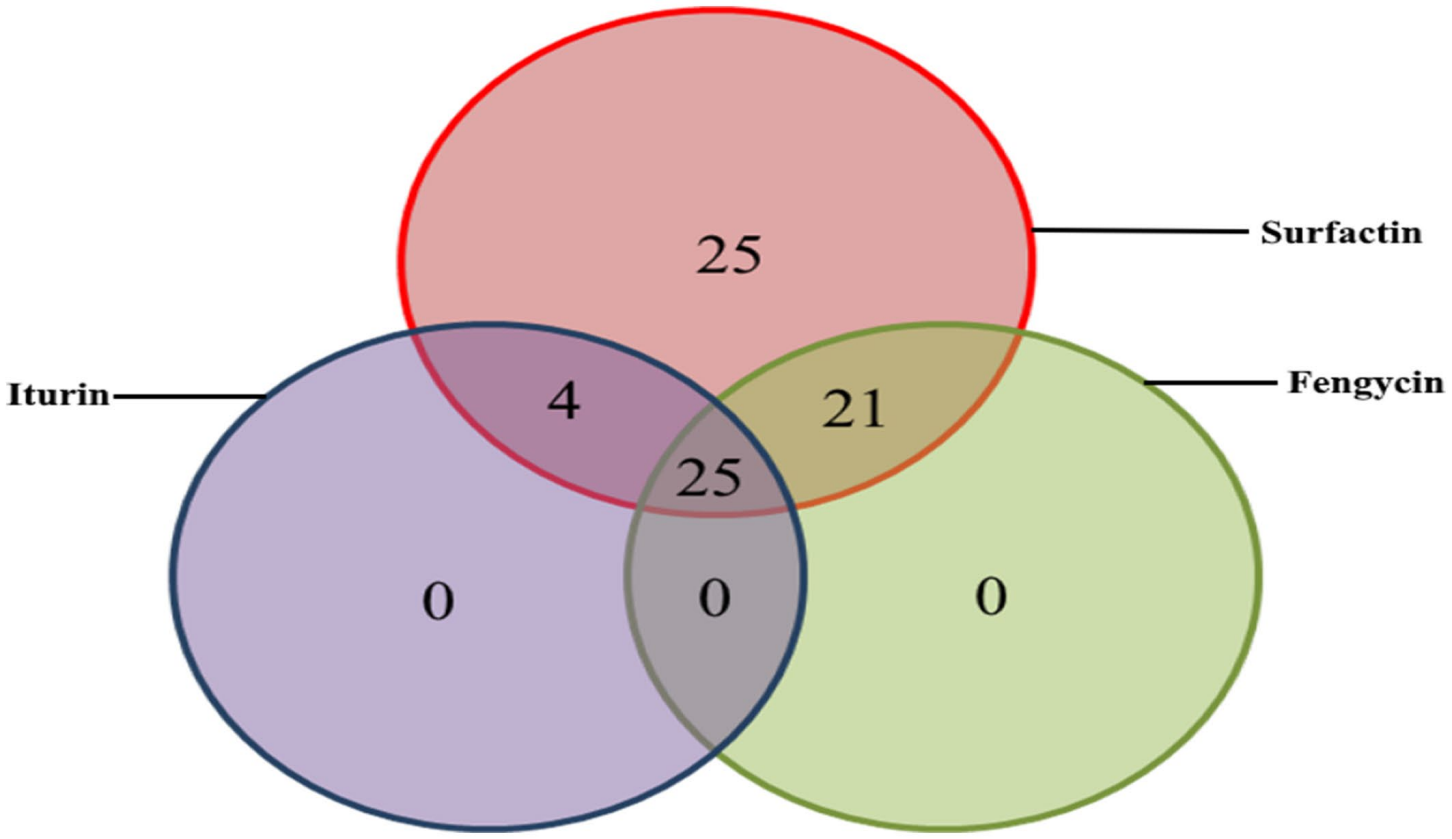
Prevalence and coexistence of lipopeptide biosynthesis gene clusters in 123 chromosomes analysis from *Bacillus* (Venn diagram, *n* = 75).

*In silico* analysis of the genomic sequences revealed the potential production of four lipopeptide families (Table 2). These are the surfactin, iturin, fengycin and kurstakin families depending on the strains. Over the 123 chromosomes analyzed, 75 (61%) possessed gene clusters responsible for surfactin production, 29 (23%) for iturin, 46 (37%) for fengycin and 19 (15%) for kurstakin (Table 3). No locillomycin synthetic gene cluster was detected in the genomes explored in this study. All chromosomes carrying kurstakin BGCs came from species belonging to the *Bacillus cereus* group. When this cluster was present, the other clusters (surfactin, fengycin, iturin) were absent. A kurstakin variant was detected in *Bacillus mycoîde* BGSC4BQ1 (Table 3, Figure 2). A variation was found in monomer 6, which is a threonine instead of Gln or Glu, which are most often found in this position. In addition, 25 chromosomes contained only clusters of surfactin genes. No chromosomes contained only fengycin or iturin gene clusters. A co-existence of gene clusters was also observed. As far surfactin and fengycin gene clusters are concerned, they were found in 21 chromosomes, while surfactin and iturin gene clusters were found in 4 chromosomes. A total of 25 chromosomes contained surfactin, fengycin and iturin gene clusters (Figure 3). In addition, six chromosomes contained four gene clusters (surfactin, fengycin, iturin, and novel gene clusters). All *B. subtilis*, *B. velezensis*, *B. licheniformis, B. paralicheniformis, B. amlyloliquefaciens, B. pumilus, B. cellulasensis, B. altitudinis, B. spizizenii*, *B. safensis, B. vallismortis, B. halotolerans, B.inaquosorum, B. stratosphericus, B. aerophilus* and *B. atrophaeus* have gene clusters to produce a known surfactin or variant. In addition, all *B. licheniformis* and *B. paralicheniformis* have gene clusters which produce lichenysin A (Table 3). All 29 iturin-producing strains belong to the *B. subtilis* group (*B. subtilis, B. velezensis, B. amyloliquefaciens, B. spizizenii, B. atrophaeus*). Gene clusters for the biosynthesis of bacillomycin D and bacillomycin L, variants of iturin were detected in the chromosomes of *B. subtilis*, *B. amyloliquefaciens* and *B. velezensis* species. In species such as *B. atrophaeus* and *B. spizizenii*, chromosomes possess gene clusters producing iturin A and mycosubtilin, respectively. For the fengycin family, A or B production gene clusters were detected in 1/3 of the chromosomes of producing strains. In fact, the 6th monomer, which may be Val in the case of fengycin B or Ala in the case of fengycin A, was not predicted during the study. A fengycin B variant was detected in the genomes of 10 strains belonging to the *B. subtilis* species (Table 3). However, all the genomes of *B. paralicheniformis* strains contained clusters of fengycin B biosynthesis genes (Table 3). The D-allothreonine observed in fengycin B is predicted to be a D-threonine by antiSMASH because these two threonine isomers can be selected by the same A-domain. A small difference is noticed in the beta carbon. Beta carbon of D-allothreonine (CαD, CβD) belongs to the D series whereas the beta carbon of D-threonine (CαD, CβL) belongs to the L series.

### 3.4 Prevalence of BGC NRPSs in chromosomes of the different species

**Figure 4:**
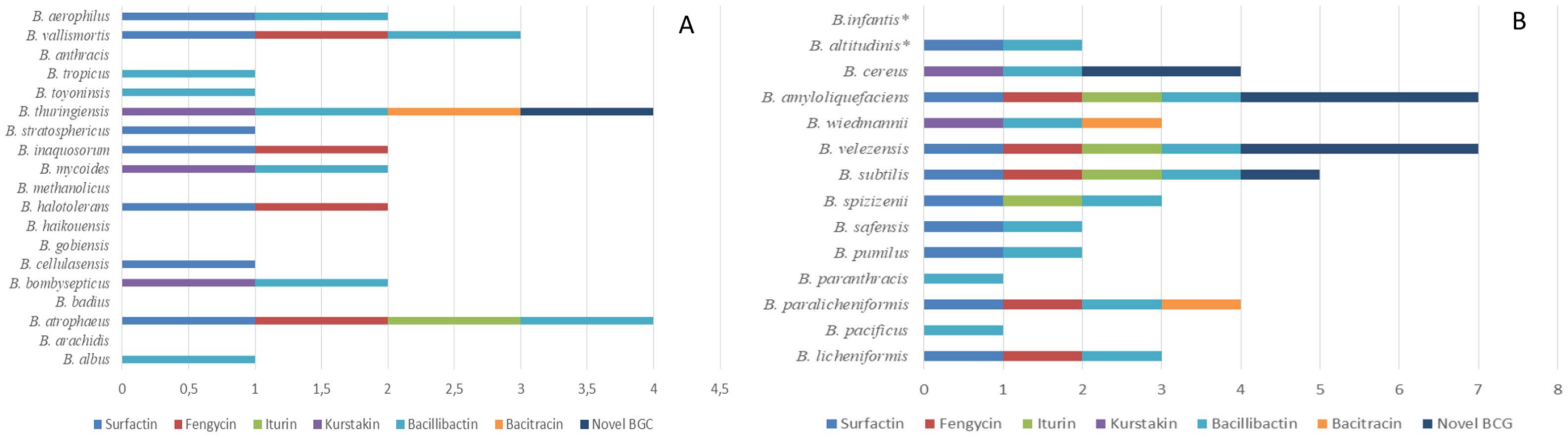
Production capacity of known BGC NRPSs and new gene clusters depending on the species of the genus *Bacillus*. Strains in **(A)** are only isolated from soil samples, but in **(B)**, apart from *B. infantis* and *B. altitudinis* which are only present in fermented food samples, the other strains can be found in soil or fermented foods. These two, which are only present in fermented food samples, are shown with asterisk in **(B)**.

The 123 strains represent 33 species with distributions as follows: 19 species were found in the soil samples only, 12 species were found in both soil and fermented food isolation media and 2 species were found only in the fermented food. *Bacillus* species that were only found in soil samples included *B. albus, B. arachidis, B. atrophaeus, B. badius, B. bombysepticus, B. cellulasensis, B. gobiensis, B. haikouensis, B. halotolerans, B. methanolicus, B. mycoides, B. inaquosorum, B. stratosphericus, B. thuringiensis, B. toyoninsis, B. tropicus, B. anthracis, B. vallismortis* and *B. aerophilus* (Figure 4A). Species found in soil and fermented foods were: *B. licheniformis, B. paralicheniformis, B. paranthracis, B. pacificus, B. pumilus, B. safensis, B. spizizenii, B. subtilis, B. velezensis, B. wiedmannii, B. amyloliquefaciens* and *B. cereus*. Two species, *B. altitudinis* and *B. infantis*, were only found in fermented foods (Figure 4B). Among genomes of soil *Bacillus* species, only *B. atrophaeus* had a gene cluster for iturin biosynthesis. Furthermore, only the genome of *B. thuringiensis* species carried a gene clusters for the biosynthesis of the antibiotic bacitracin and a new gene cluster. Analysis of genomes of the 12 species found in isolation media (soil and fermented food) revealed the presence of new gene clusters in species such as *B. velezensis*, *B. amyloliquefaciens*, *B. subtilis* and *B. cereus*. Prevalence of new gene clusters was higher in *B. velezensis* and *B. amyloliquefaciens*, respectively (Figure 4B) and the genomes of these two strains carried 3 new BGC NRPSs. The genome of *B. infantis*, a species that was only present in fermented foods had no NRPSs gene cluster (Figure 4B). Moreover, bacitracin biosynthesis genes were carried only by the genomes of *B. paralicheniformis* and *B. thuringiensis*. Genomes of *B. pacificus*, *B. cereus*, *B. wiedmannii* and *B. paranthracis* lacked surfactin biosynthesis genes (Figure 4).

### 3.5 Bacitracin production

Among the 123 genomes screened, only 4 (*B. paralicheniformis* 14DA11, *B. paralicheniformis* CP47, *B. paralicheniformis* UBBLI-30, and *B. thuringiensis* Bt185) bear BGC corresponding to the synthetic pathway for antibiotic bacitracin. This antibiotic is a semi-cyclic peptide constituted of 12 amino acids. In *B. paralicheniformis* CP47 chromosome sequence, a BGC encoding an NRPS containing 12 modules was found. These 12 modules correspond exactly to the 12 modules of bacitracin synthetase. In the genomes of *B. thuringiensis* Bt185, *B. paralicheniformis* 14DA11, and *B. paralicheniformis* UBBLI-30, modular structuring and amino acid composition were similar to bacitracin A1 (Figure 1), similarities ranged from 85 to 100%.

### 3.6 BGC potentially producing new metabolites

A total of 7 new molecules carried by 7 new gene clusters were detected in this study, based on the exploration of 123 complete genomes of strains belonging to the *Bacillus* genus and isolated either from fermented foods or from soil. These newly identified gene clusters would be capable of producing other new lipopeptide families and new antibiotics. All newly predicted molecules were first compared with those identified subsequently, and then with the non-ribosomal peptides available in the Norine database. Low similarities ranged from 28.6 to 50%. The first new gene cluster detected consisted of 3 genes and 4 modules incorporating Thr, X, Asn and Asn monomers, respectively (Figure 2). Amino acid incorporated by module 2 was not detected, hence the letter X at this position (Figure 2). The predicted tetrapeptide showed a low similarity of 39.4% to Cis-7-tetradecenoyl-D-Asparagine. This new gene cluster was carried by plasmids from *B. cereus* CMCC P0011 and *B. cereus* CMCC P0021 strains. Two new gene clusters consisting of 6 modules each were detected by exploring the genomes of *B. velezensis* ATR2, *B. velezensis* DSYZ, *B. velezensis* CGMCC 11640 and *B. amyloliquefaciens* WF02. The hexapeptide predicted in the *B. velezensis* ATR2 genome was structured as follows: D-Cys-Ser-Cys-Ala-X-D-Asn, and contained a CAL (Co-enzyme A ligase) domain. The hexapeptide predicted in the genomes of *B. velezensis* DSYZ, *B. velezensis* CGMCC 11640 and *B. amyloliquefaciens* WF02 was structured: Val-D-Phe-Asp-D-Asn-Gly-D-Glu. Formation is ensured by the combination of an NRPS system and a PKS (polyketide synthase) system (Figure 2). CAL and C-starter domains were not detected in this hexapeptide. Therefore, it is probably an antibiotic. Literature searches and structural analyses in Norine did not reveal any similar molecules.

*In silico* analyses also revealed the presence of 3 new NRPS clusters capable of producing 3 different heptapeptides depending on the modular organization and amino acid composition of each. The first predicted heptapeptide detected was carried by *B. velezensis* DSYZ and *B. subtilis* SJ-10 genomes and is structured as follows: D-Cys-Ser-Cys-Ala-X-Asn-D-Asn (Figure 2). Monomer 5 has not been predicted. A CAL domain was detected at the start of the peptide chain (Figure 2). Structural analysis of this heptapeptide in Norine showed little similarity to iturin A1 (32.3%). Furthermore, literature searches revealed no molecules with a similar structure. For the last two predicted heptapeptides, each gene cluster consists of 7 modules incorporating 7 amino acids. One had the following structure: D-Phe-D-Leu-Phe-D-Thr-Val-Ala-Thr and was carried by the *B. amyloliquefaciens* HM618 genome while the other had the following structural architecture: D-Phe-Tyr-Ile-X-D-Phe-Leu-Leu and was carried by the *B. anthracis* CMF9 genome (Figure 2). Their structures were similar to kahalalide A (39.4%) and axinastatin 5 (40%) respectively. The heptapeptide identified in the *B. amyloliquefaciens* HM618 genome contained a thioesterase (TE) domain marking the end of the peptide chain. The heptapeptide identified in the *B. anthracis* genome contained a CAL domain at the start of the peptide chain and a C condensation domain at the end of module 7 (Figure 2).

Exploration of the plasmid sequence of *B. cereus* CMCCPOO11 and *B. cereus* CMCCPOO21 revealed a BGC probably responsible for the synthesis of a new octapeptide nonribosomal peptide (Figure 2). The chemical structure of unpredictable monomers are represented by the letter X in the peptide chain. The structure search on Norine showed little similarity (50%) with cyanostatin B. No molecules with a similar structure were found in the literature.

## 4 Discussion

*Bacillus* species are known to produce a large variety of secondary metabolites. This production is influenced by environmental conditions. Thus, this study explored 123 complete genomes of *Bacillus* isolated from soil and fermented foods. Thus, *in silico* analysis of chromosomal and plasmid sequences using bio-informatics tools specific to non-ribosomal peptides revealed a potential gene cluster responsible for the biosynthesis of lipopeptides (sufactin, fengycin, iturin, kurstakin), the antibiotic bacitracin and the siderophore bacillibactin. Only *B. subtilis* 916 (Luo et al., 2015a) produced locillomycin, which was not detected in this study. However, surfactins are lipoheptapeptides with variants such as esperin, lichenysin, pumilacidin and surfactin (Waongo et al., 2023). Two distinct amino acids were predicted to be incorporated in the first module: either a glutamate (Glu) or a glutamic acid (Gln) (Hu et al., 2019). As for iturins, they are lipoheptapeptides whose main variants are: iturins A and C, bacillomycins D, F and L, mycosubtilin and mojavensin (Waongo et al., 2023). Bacillibactin is a siderophore whose sequence is Dhb-Gly-Thr (May et al., 2001). As a result, the modular organization and monomer composition of predicted peptides from the surfactin and iturin groups as well as bacillibactin, were similar to literature data. Compared with fengycin, the lipodecapeptide predicted in genomes of *B. subtilis* UD1022, *B. vallismortis* DSM11031, *B. subtilis* ZD01, *B. halotolerans* ZB201702, *B. mojavensis* B-41812, *B. mojavensis* B-41341, *B. subtilis* MEC_B298, *B. subtilis* s-16, *B. subtilis* FUA2231, *B. subtilis* FUA2232, *B. subtilis* SRCM103517, and *B. subtilis* BSP1, contains at position 8 a glutamic acid (Glu). In contrast, various authors have reported that module 8 incorporates glutamine (Gln) during peptide chain formation (Ait Kaki et al., 2020; Hussein, 2019). This observed variability shows that certain *Bacillus* could produce a fengycin B variant. Also, structural analysis of predicted kurstakins shows the presence of a threonine (Thr) at position 6 in the *B. mycoide* BGSC 4BQ1 genome. Generally, the amino acid occupying this position in the case of kurstakin is glutamine (Gln) (Béchet et al., 2012). Although monomers 2 and 6 have not been predicted in the genomes of other *B. cereus* group species (*B. thuringiensis*, *B. cereus*, *B. wiedmannii*, *B. bombysepticus*), it is quite possible that *B. mycoide* BGSC 4BQ1 would be capable of producing a kurstakin variant. Nevertheless, the two putative variants identified through this *in silico* screening approach will need to be further investigated through *in vitro* experiments.

This study revealed the coexistence of lipopeptide biosynthesis genes. Indeed, Luo et al. (2015b) reported the coexistence of surfactin (sfr), bacillomycin (bmy), fengycin (fen) and locillomycin (Loc) gene clusters in *B. subtilis* 916 genome. In this study, *B. amyloliquefaciens* WF02, *B. amyloliquefaciens* HM618, *B. velezensis* ATR2, *B. velezensis* CGMCC 11640, *B. velezensis* Lzh-a42, *B. subtilis* SJ-10 and *B. velezensis* DSYZ genomes also contained three known lipopeptide biosynthesis gene clusters and novel gene cluster that could produce a new lipopeptide family. To our knowledge, no study has demonstrated the coexistence of four or five gene clusters of lipopeptide in genomes of species such as *B. amyloliquefaciens* and *B. velezensis*. According to Luo et al. (2015b), a co-production of surfactin, fengycin, iturin and locillomycin by *B. subtilis* 916 is at the origin of its inhibitory capacity against multi-resistant *Staphylococcus aureus*. Similarly, the co-production of lipopeptides would reduce hemolytic activity of the producing strains (Luo et al., 2019; Waongo et al., 2023).

The emergence of multi-resistant strains to commonly used antibiotics is a major challenge (Eduardo-Correia et al., 2020). The exploration of new molecules that could serve as alternatives remains a necessity. Thus, the study identified seven new gene clusters (NRPS and NRPS/PKS), two of which were carried by plasmid sequences. These new gene clusters were responsible for the biosynthesis of a tetrapeptide, two hexapeptides, three heptapeptides and octapeptide. The predicted tetrapeptide and octapeptide were synthesized by gene clusters carried by plasmids. The structural architecture and monomer composition of all predicted molecules differed from the peptides available in the database (Flissi et al., 2023). Furthermore, literature searches did not reveal any molecules with similar structure and monomer composition. Except for the hexapeptide predicted in the chromosomes of *B. velezensis* DSYZ and *B. amyloliquefaciens* WF02, all the others carried a CAL or C-starter domain in the first module. In fact, lipopeptides are made up of chains of amino acids and fatty acids. Their structure is characterized by the presence of C-starter and CAL domains, which play a role in fatty acid chain activation (Ongena and Jacques, 2007). Thus, their presence in the first module of the seven predicted new peptides means that they could belong to the lipopeptide family. However, certain antibiotics of purely peptidic clinical interest, such as vancomycin and bacitracin, are characterized by the presence of an adenylation domain (A) in the first module (Konz et al., 1997; Wageningen et al., 1998). This A domain is responsible for selecting and activating the first amino acid to be integrated into the growing peptide chain. Consequently, the new hexapeptide predicted in the chromosomes of *B. velezensis* DSYZ and *B. amyloliquefaciens* WF02 would be a peptide antibiotic. Esmaeel et al. (2016) reported the synthesis of a new lipopeptide by analyzing the genomes of genus *Burkholderia*. On the other hand, since lipopeptides are synthesized non-ribosomally, the presence of gene clusters responsible for their synthesis in plasmids is rare. According to the literature, no study has revealed the presence of NRPS lipopeptide synthase genes on a *Bacillus* plasmid, only on the *Burkholderia* plasmid (Esmaeel et al., 2016). As a result, the putative new BGCs would constitute new families of lipopeptides as well as new antibiotics, which could have unique and interesting biological properties (e.g., antifungal, antibacterial, antiviral). Biomolecules newly predicted in this study could be at the center of current research in the interest of their future use in several fields such as agri-food, cosmetics and medicine. The results of this study reveal that the genomes of *Bacillus* strains available in databases contain many unknown molecules that could play important roles in antimicrobial control. However, it would be interesting to elucidate the biological functions of all these new molecules. Although the bioinformatic discovery of novel molecules often captures attention, rigorous experimental validation remains the crucial step in translating promising ideas into tangible scientific findings and ensuring genuine progress.

## 5 Conclusion

Exploration of microorganisms genomes allows for the rapid identification of gene clusters located on chromosomes coding for new beneficial molecules. Thus, in addition to chromosomes, it would be interesting to analyze plasmids that could have BGC clusters of NRPs or lipopeptides. Then, this study allowed us to identify seven new gene clusters synthesizing new non-ribosomal peptides. Our results suggest that several NRPs capable of being produced by *Bacillus* strains isolated from fermented foods and soil are still uncharacterized and their properties still unknown. Therefore, targeted research should be conducted experimentally to validate these different predicted molecules.

## Data Availability

Genome mining work was done base on the whole genome sequences of 123 *Bacillus* strains is listed in Table 1, which can be obtained from NCBI nucleotide database (https://www.ncbi.nlm.nih.gov/nuccore). All the other data supporting the findings is contained within the manuscript.

## Footnotes

^1^ https://www.ncbi.nlm.nih.gov/nuccore

^2^ https://bioinfo.cristal.univ-lille.fr/norine
